# Antihypertensive Therapy by ACEI/ARB Is Associated With Intestinal Flora Alterations and Metabolomic Profiles in Hypertensive Patients

**DOI:** 10.3389/fcell.2022.861829

**Published:** 2022-03-23

**Authors:** Ying Dong, Pan Wang, Jie Jiao, Xinchun Yang, Mulei Chen, Jing Li

**Affiliations:** Heart Center and Beijing Key Laboratory of Hypertension, Beijing Chaoyang Hospital, Capital Medical University, Beijing, China

**Keywords:** ACEI/ARB, anti-hypertensive, hypertension, intestinal flora, metabolomics

## Abstract

Angiotensin-converting enzyme inhibitors and angiotensin receptor blockers (ACEI/ARB) are the first-line drugs for the treatment of essential hypertension (HTN), one of the most important risk factors for cardiovascular and cerebrovascular diseases. Intestinal flora and microbial metabolites have been demonstrated to play important roles in blood pressure (BP) regulation and HTN development. However, it remains elusive that intestinal bacteria and metabolites are associated with the protective effects of ACEI/ARB anti-hypertensive drugs against HTN. In this study, we evaluated the effect of ACEI/ARB on gut microbiome and metabolites in patients suffering from HTN. We performed 16S rRNA sequencing and fecal metabolomic analysis of 36 HTN patients placed on ACEI/ARB therapy and 19 newly diagnosed HTN patients with no history of anti-hypertensive treatment. Patients under medication treatment were further classified into well-controlled (*n* = 24) and poor-controlled (*n* = 12) groups according to their BP levels. The ACEI/ARB improved the intestinal microbiome of the HTN patients by reducing potentially pathogenic bacteria such as *Enterobacter* and *Klebsiella* and increasing beneficial bacteria such as *Odoribacter*. Moreover, ACEI/ARB therapy was correlated with significant metabolomic changes in the HTN patients, including progressively enhanced inositol from poor-controlled to well-controlled groups. The profiles of gut bacteria were linked to the production of metabolites, and inositol was negatively correlated with *Klebsiella*, *Enterobacter*, and *Proteobacteria*. Our study suggests that ACEI/ARB modulates gut microbial composition and functions and alters microbial metabolites in HTN patients.

## Introduction

Hypertension (HTN), also known as high blood pressure (BP), is one of the most prevalent chronic diseases. According to the WHO, more than 1.2 billion adults aged 30–79 years have HTN, and most live in low- and middle-income countries (World Health Organization, 2021). Recent studies have suggested that intestinal microbiota dysbiosis and dysfunction have an important role in high BP regulation and HTN pathogenesis. We have previously reported the striking difference in gut microbial composition, gene functions, and metabolic profiling between hypertensive individuals and healthy controls (Li et al., 2017). Moreover, a recent study found a specific shift of intestinal flora metabolome, serum metabolome, and urine metabolome in hypertensive patients compared to healthy controls (with normal BP) (Huart et al., 2019). In addition, a cohort study focusing on intestinal flora and plasma metabolomics of 1,082 Chinese adults without medications has further uncovered the relationship between serum metabolites and the host's BP, demonstrating that a specific bacterial community and metabolites are positively correlated with the host's BP levels (Wang et al., 2021).

Guideline-based approaches to pharmacotherapy have been widely adopted to simplify its clinical practice and improve BP control (Whelton et al., 2018; Williams et al., 2018). Angiotensin-converting enzyme inhibitors and angiotensin receptor blockers (ACEI/ARB), as the first-line of medical treatment for HTN, have shown to be beneficial against gastrointestinal symptoms, such as abdominal pain, diarrhea, and nausea (Tan et al., 2020). Moreover, some studies have shown that these drugs can reduce the risk of colorectal cancer (Cheung et al., 2020). In addition, some studies proposed that anti-hypertensive medicines may affect intestinal microbiota (Li et al., 2017; Yan et al., 2017; Wang et al., 2021). Yet, the effect and mechanism of ACEI/ARB on intestinal flora and microbial metabolite characteristics in hypertensive individuals are not fully understood.

Given a piece of clue reported most recently that valsartan administration is associated with the intestinal microbiota of spontaneously hypertensive rats (Qi et al., 2021), in this study, we hypothesized that ACEI/ARB anti-hypertensive drugs might elicit dramatic improvements in the gut microbiome and metabolites of patients suffering from HTN.

## Materials and Methods

### Study Population

A total of 55 HTN patients were enrolled at Beijing Chaoyang Hospital between January and May 2021. HTN was defined as systolic BP (SBP) ≥140 mmHg, and/or diastolic BP (DBP) ≥90 mmHg, and/or previous history of HTN, and/or use of anti-hypertensive medicine before enrollment according to the 2010 Chinese guidelines for the management of HTN (Liu, 2011). The measurement of BP was based on serial data. Such as previous history of HTN and/or the use of anti-hypertensive medicine before enrollment, according to the 2010 Chinese guidelines for the management of HTN (Liu, 2011). The BP of each participating patient was measured in their sitting position, three times before their enrollment, by physicians with a random-zero mercury column sphygmomanometer and the fecal and serum samples collected. The BP of the patients was measured once randomly, and twice in the mornings. The BP was acquired on two consecutive days. The average data of these three times were used as the final BP. The exclusion criteria were: 1) hypertensive patients who received other anti-hypertensive drugs, such as calcium channel blocker and beta blockers; 2) patients suffering from cancer, heart failure, renal failure, stroke, peripheral artery disease, and chronic inflammation; 3) subjects who received statin, aspirin, insulin, metformin, etc.; and 4) individuals who were prescribed antibiotics or were using probiotics over the past two months.

Hypertensive patients were classified into the following categories: untreated group, i.e., newly diagnosed HTN patients who had never received anti-hypertensive treatment (*n* = 19), and treated group, i.e., those HTN patients who were under ACEI/ARB treatment (*n* = 36) for at least 4 weeks. In addition, the HTN patients treated with ACEI/ARB were further divided into the well-controlled group (*n* = 24, SBP <140 mmHg and DBP <90 mmHg) and poor-controlled group (*n* = 12, SBP ≥140 mmHg or DBP ≥90 mmHg) according to the hosts' BP levels.

The protocol of this study was approved by the Medical Ethics Committee from Beijing Chaoyang Hospital. Written informed consent was obtained from all patients prior to enrollment.

### Fecal Sample Collection and 16S rRNA Sequencing

Fresh fecal samples were collected with individual sterile EP tubes, then quickly frozen and stored at −80°C in a cryogenic freezer for cryopreservation. The bacterial DNA was extracted using the TIANGEN kit according to the manufacturer’s instructions. The concentration and quality of the extracted DNA were photometrically determined with a NanoDrop^®^ ND-2000c UV–vis spectrophotometer (NanoDrop Technologies, Wilmington, DE, United States). The qualified DNA from the fecal samples was diluted to 1 ng/μl and subjected to the amplification of polymerase chain reaction with a universal primer set directed at the hypervariable V4 region of the bacterial 16S rRNA gene (515F 5′-GTGCCAGCMGCCGCGGTAA-3′ and 806R 5′-GGACTACNNGGGTATCTAAT -3′) and Phusion^®^ High-Fidelity PCR Master Mix with GC Buffer (New England Biolabs Co., Ltd.). PCR products were detected by electrophoresis using 2% agarose gel, and the target bands were recovered using gel recovery kits provided by QIAGEN. Following the 16S rDNA library, preparation, and generation with a TruSeq^®^ DNA PCR-Free Sample Preparation Kit, the quality of the library was assessed by using the Qubit@2.0 fluorometer (Thermo Scientific) and the Agilent Bioanalyzer 2100 system, and sequenced by Illumina NovaSeq6000.

### Operational Taxonomic Units Clustering and Taxon Annotation

Based on the unique barcode, raw reads were assigned to different samples, and the assigned paired-end reads of each sample were merged to raw tags by FLASH (Version 1.2.7). The raw tags were filtered, after which the clean tags were obtained using the QIIME (Version 1.9.1) quality control process. Clean tags were aligned to the Gold database, and the chimera sequence was detected by the UCHIME algorithm. Finally, the non-chimera clean tags were considered effective tags, which were clustered into operational taxonomic units (OTUs) with ≥97% sequence identity; chimeras were removed by using Uparse (Version 7.0.1001). Sequences with the highest frequency in the OTUs were selected as representative sequences of the OTUs and applied for taxonomic annotation by the RDP classifier according to the Greengene database.

### Alpha Diversity of Fecal Microbiota

Alpha diversity was performed to analyze the diversity and richness of the gut microbial community within the sample. QIIME software (Version 1.9.1) was applied to access bacterial alpha diversity parameters, including observed OTUs, Chao1 values, and Shannon and Simpson indices. Rarefaction curves were carried out by randomly extracting a certain amount of sequencing data from the samples, calculating the number of OTUs, building a curve based on the amount of sequencing data extracted, and the corresponding OTU numbers. The rarefaction curves directly reflected the rationality of the sequencing data and could indirectly reflect the richness of the OTUs in the samples. When the curve tends to be flat, it indicates that the sequencing data amount is progressive and reasonable, and more data would generate few novel OTUs.

### Beta Diversity of Gut Microbiota

Beta diversity is a comparative analysis of the microbial community composition of different samples. According to the OTU abundance of all the samples, the systematic relationship between the OTUs was used to analyze the UniFrac distance, indicating the distance between the samples by the information between the microbial sequences in each sample. The multivariate statistical methods included principal component analysis (PCA) and principal coordinate analysis (PCoA). The PCA was obtained through ade4 and ggplot2 packages and PCoA through the WGCNA, stats, and ggplot2 packages in the R software (version 3.3.3).

### Microbial Dysbiosis Index

The microbial dysbiosis (MD) was determined as previously described (Xu et al., 2020). To access the degree of gut microbial dysbiosis, the MD index was analyzed as the log of [total abundance of organisms increased in untreated patients]/[total abundance of organisms reduced in well-controlled patients] for all the samples.

### Functional Annotation

Prediction for the gut microbial community’s functional capabilities was performed by the phylogenetic investigation of communities by reconstruction of unobserved states (PICRUSt) Version 1.0.0 to generate the Kyoto Encyclopedia of Genes and Genomes (KEGG) ontology profiles. Briefly, based on the tree of the OTUs in the Greengene database and the gene information in the OTUs, the gene function of their shared ancestor was inferred through the KEGG database. Thus, the composition of the bacterial community was mapped to the database, constructing the gene function prediction of the gut bacteria.

### Gas Chromatography–Time of Flight Mass Spectroscopy

In the present study, serum samples from 55 patients were subjected to a metabolomics analysis based on untargeted gas chromatography–time of flight mass spectroscopy (GC-TOF/MS). Overnight fasting venous blood samples were collected the next morning. The whole blood samples were then separated into a serum by centrifugation at 4°C. Then, 50 μl of the sample was mixed with 10 μl of the internal standard, and 175 μl of pre-chilled methanol/chloroform was added. After centrifugation at 14,000 *g* and 4°C for 20 min, 200 μl of the supernatant was transferred to an autosampler vial and subjected to GC-TOF/MS analysis. The untargeted metabolomics profiling was performed on the XploreMET platform (Metabo-Profile, Shanghai, China) and an untargeted GC-TOF/MS system with Agilent 7890B gas chromatography and a Gerstel multipurpose sample MPS2 (Gerstel, Muehlheim, Germany) were applied. For separation, an Rxi-5 ms capillary column (inner diameter: 30 m × 250 μm, film thickness: 0.25 μm; Restek Corporation, Bellefonte, PA, United States) was used. Helium at a constant flow rate of 1 ml/min was the carrier gas. The injection and transfer interfaces were at a temperature of 270°C, while the temperature for the source was at 220°C. The measurement was performed in the full scan model (m/z 50–500) at electron impact ionization (70 eV).

### Spectra Data Preprocessing and Metabolite Annotation

The raw mass spectral data generated by GC-TOF/MS were processed on the XploreMET software (v3.0, Metabo-Profile, Shanghai, China) to denoise and smoothen, pick peaks, and align metabolite signals. Data sets were transformed into data vectors for statistical analysis. All the measurements were expressed as the mean and standard deviation of the observed peaks. Using the XploreMET software, the retention indices and mass spectral data were compared with those reference standards of known chemicals presented in the JiaLib metabolite database, comprising over 1,200 mammalian metabolites purchased from Sigma-Aldrich (St. Louis, MO, United States), Santa Cruz (Dallas, TX, United States), and Nu-Chek Prep (Elysian, MN, United States). Thus, the precise metabolite annotation in each sample was performed.

### Multivariate Statistical Analyses of Metabolome

The PCA serves as an unsupervised model to detect outliers, clustering, and classification trends. The principal components derived from the data set indicate the orthogonality to one another and reflect the reducing levels of the variations. The PCA significantly decreases the number of variables required for data representation and provides visualization of data clusters and outliers based on multiple variables. Partial least-square discriminant analysis (PLS-DA) and orthogonal PLS discriminant analysis (OPLS-DA) were used for multiclass classification, thus providing more valuable information beyond that obtained from the PCA. These are sophisticated multivariate statistical models for mass spectral data and class variables that can detect the differences in the metabolic profiles between groups. Statistical analyses of PCA, PLS-DA, and OPLS-DA were carried out with the software packages in R Studio (R Foundation for Statistical Computing, Vienna, Austria).

### Statistical Analyses

Continuous variables were presented as the median with an interquartile range. The categorical variables are shown as numbers and percentages. The Wilcoxon rank test and chi-squared tests were used to examine continuous and categorical variables, respectively. Student’s *t*-test or the Mann–Whitney Wilcoxon test was used to determine the significant difference between the groups. The linear discriminant analysis (LDA) effect size (LEfSe) version 1.0 was performed to identify taxa and KEGG pathways that are significantly different between the groups, with an LDA score = 2 as the cutoff value. The Z-score in the heat map was obtained by subtracting the mean and dividing the standard deviation. The Z-score was negative when the raw value was lower than the mean and was positive when higher than the mean. A volcano plot was applied for depicting significantly different variables between the groups. The analyses were carried out using SPSS, Version 22.0 (IBM Corp. Armonk, New York). In addition, a different abundance of genera and functions was determined by the Wilcoxon rank-sum test. The presented *p* values are two-tailed, and a *p*-value <0.05 was considered statistically significant.

## Results

### Characteristics of the Hypertensive Patients

The present study included 19 untreated hypertensive patients with no history of anti-hypertensive treatment and 36 HTN subjects under ACEI/ARB therapy (including 24 well-controlled and 12 poor-controlled). The primary characteristics of the patients (*n* = 55) are shown in Supplementary Table S1. Compared with untreated or poor-controlled patients, the SBP and DBP levels were significantly lower (*p* < 0.05) in the subjects showing well-controlled status under ACEI/ARB therapy. Moreover, except for age, there was no difference between groups in sex, cigarette smoking, body mass index, fasting blood glucose, total cholesterol, LDLC, and uric acid and creatinine. Yet, the average age was 49 years in the well-controlled group, and 43.5 years in the poor-controlled group, which is consistent with a previous study (Wang et al., 2014), suggesting that the control rate of BP in HTN patients was higher among the elderly in China.

### Gut Microbial Diversity in Hypertensive Patients Treated With Angiotensin-Converting Enzyme Inhibitors and Angiotensin Receptor Blockers

In total, 6,115,624 high-quality clean tags with an average of 99,219 reads per sample were obtained for the fecal microbiota analysis. A shift in the microbial diversity is considered as a crucial factor indicating the degree of gut dysbiosis associated with diseases (Bäckhed et al., 2015). Moreover, alteration in the fecal microbiota structure has been found in HTN patients when compared to healthy individuals (Li et al., 2017). This study further examined alterations in the microbiome between HTN patients who received ACEI/ARB treatment and those who did not. No differences in OTUs, or alpha diversity indices (diversity within samples), including the Chao1, Shannon, and Simpson indices, were found between the groups (Supplementary Figure S1A). Yet, there was a trend toward elevated microbial alpha diversity in treated patients, but the difference was not significant.

Moreover, hypertensive patients who achieved the desired BP levels by ACEI/ARB treatment or without were further examined. No differences in the OTU numbers, Chao1 richness, Shannon index, or Simpson index were observed among the untreated, poor-controlled, and even well-controlled groups (Supplementary Figure S1B). However, the well-controlled groups showed an increasing tendency in the gut microbial diversity without statistic differences. Thus, it is suggested that ACEI/ARB therapy might exert protection against gut dysbiosis in hypertensive patients to a slight extent but fail to completely restore the disordered situation in alpha diversity.

Furthermore, to evaluate the features and structures of the intestinal microbiota in the hypertensive patients, beta diversity by the PCA and PCoA plots based on the phylum, class, order, family, and genus levels was conducted and compared, respectively. Neither PCA nor PCoA analysis could distinguish the untreated and treated patients into different clusters (Supplementary Figure S2). Well-controlled and poor-controlled subjects in the PCA and PCoA plots could not be distinguished from the other groups (Supplementary Figure S3). Although patients in the well-controlled group showed much lower BP levels compared with the untreated and poor-controlled patients, the impairment in both the gut microbial alpha diversity and beta diversity of HTN could not be reversed by ACEI/ARB therapy. Thus, we speculated that the influence of ACEI/ARB exerted on the microbial diversity was not strong enough to affect gut dysbiosis in HTN patients but may still alleviate dysfunction to some extent.

### Evaluation of Taxonomic Profiles Upon Angiotensin-Converting Enzyme Inhibitors and Angiotensin Receptor Blockers Interventions

**FIGURE 1:**
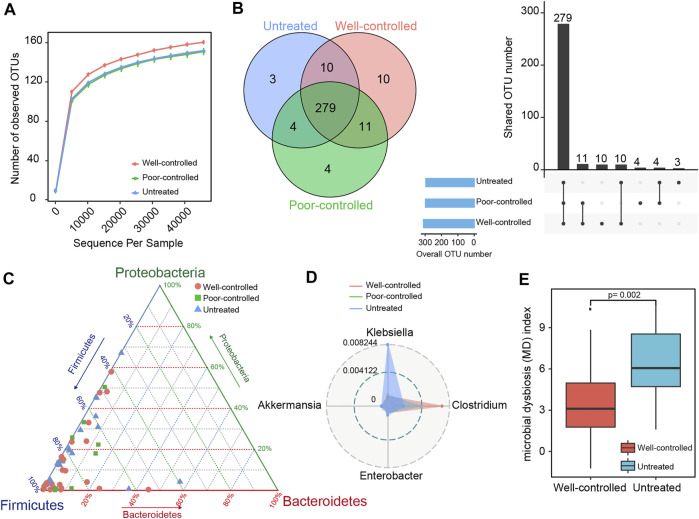
Taxonomic distribution of fecal microbiome in ACEI/ARB well-controlled and poor-controlled HTN subjects. **(A)** A rarefaction curve in each group was obtained by gradually expanding the sequencing depth of random samples. The curves approached saturation as the sample sequencing depth advanced and indicated that the sequencing data were sufficient and stable. **(B)** Venn diagrams and an upset view showing the number of OTUs shared or uniquely distributed in the untreated and treated patients (well-controlled or poor-controlled groups). The black bar charts represent the number of OTUs shared in the groups, while those at the bottom left show the number of OTUs in each group. The dotted line at the right bottom indicates the share pattern. A total of 279 OTUs were shared among groups. **(C)** The distribution of hypertensive subjects in groups is plotted into a ternary diagram based on the relative abundance of the three most dominant taxon at the phylum level: Proteobacteria, Firmicutes, and Bacteroidetes. **(D)** Radar charts for the distinct taxonomical composition of groups in the top four most abundant genera. The inner blue circle represents a relative abundance at 0.004122 and the outer gray circle represents a relative abundance of 0.008244. The untreated group was more predominant with Klebsiella, while the ACEI/ARB-treated groups were more enriched with Clostridium. **(E)** Box plots showing the gut MD index in untreated and well-controlled hypertensive patients. The boxes represent the interquartile ranges, the inside line represents the median, and the circles represent outliers. The MD index is significantly depressed in the well-controlled group as compared with the untreated ones; *p* = 0.002; Wilcoxon rank-sum test.

The rarefaction plots were assessed to characterize the sequencing data obtained from the samples. The rarefaction curves based on all the OTUs were flattened and reached a plateau in each group, implying the adequacy of sequencing depth (Figure 1A). We observed almost overlapping curves and fewer OTUs in the untreated and poor-controlled patients. More OTUs were obtained from the well-controlled patients, suggesting more abundant gut microbiota. A Venn diagram was performed to evaluate whether the pattern of shared and unique OTUs existed among the groups (Figure 1B). There were 279 OTUs overlapping in the 3 groups, and 10 OTUs were shared by the untreated and well-controlled groups. Eleven OTUs were shared by the ACEI/ARB treatment patients, regardless of being poorly controlled or well controlled, and four OTUs were shared by the untreated and poor-controlled groups. Additionally, three OTUs were uniquely presented in the untreated group, ten OTUs in the well-controlled group, and four OTUs in the poor-controlled group.

The compositions and taxonomic constitution of the fecal microbiota were then assessed at different taxonomic levels. Overall, 9 phyla, 15 classes, 21 orders, 41 families, and 60 genera were annotated. Supplementary Figure S4 shows the top 10 most enriched taxon identified in each group. The phylum Firmicutes dominated the intestinal microbiota, followed by Proteobacteria. Clostridia, Gammaproteobacteria, Bacteroidia, and Bacilli were the most predominant classes; Clostridiales and Enterobacteriales were the predominant orders; Lachnospiraceae, Ruminococcaceae, and Enterobacteriaceae were the predominant families; and Blautia, Faecalibacterium, Ruminococcus, Bacteroides*,* and Bifidobacterium were the predominant genera (Supplementary Figure S4).

For the three most dominant phyla consisting Firmicutes, Proteobacteria, and Bacteroidetes, the ternary plots in Figure 1C show that the average relative abundance of Firmicutes in the well-controlled group (75.255%) and poor-controlled group (77.039%) were higher than that of the untreated group (69.013%). The relative abundance of Proteobacteria and Bacteroidetes in the untreated group (19.706 and 6.298%, respectively) was higher than in the other groups (14.909% in poor-controlled and 12.317% in well-controlled for Proteobacteria; 4.465% in poor-controlled and 5.967% in well-controlled for Bacteroidetes). At the genus level, *Klebsiella* was predominant in the untreated group, while *Clostridium* was dominant in the ACEI/ARB group, especially in well-controlled individuals (Figure 1D). To assess the degree of fecal microbial dysbiosis, the MD index evaluating diseased status was included. Intriguingly, comparing the patients without intervention, the MD index was significantly attenuated in hypertensive subjects with well-controlled BP following ACEI/ARB treatment (Figure 1E).

### Fecal Microbiota Discriminating Hypertensive Patients From Those Under Therapy

**FIGURE 2:**
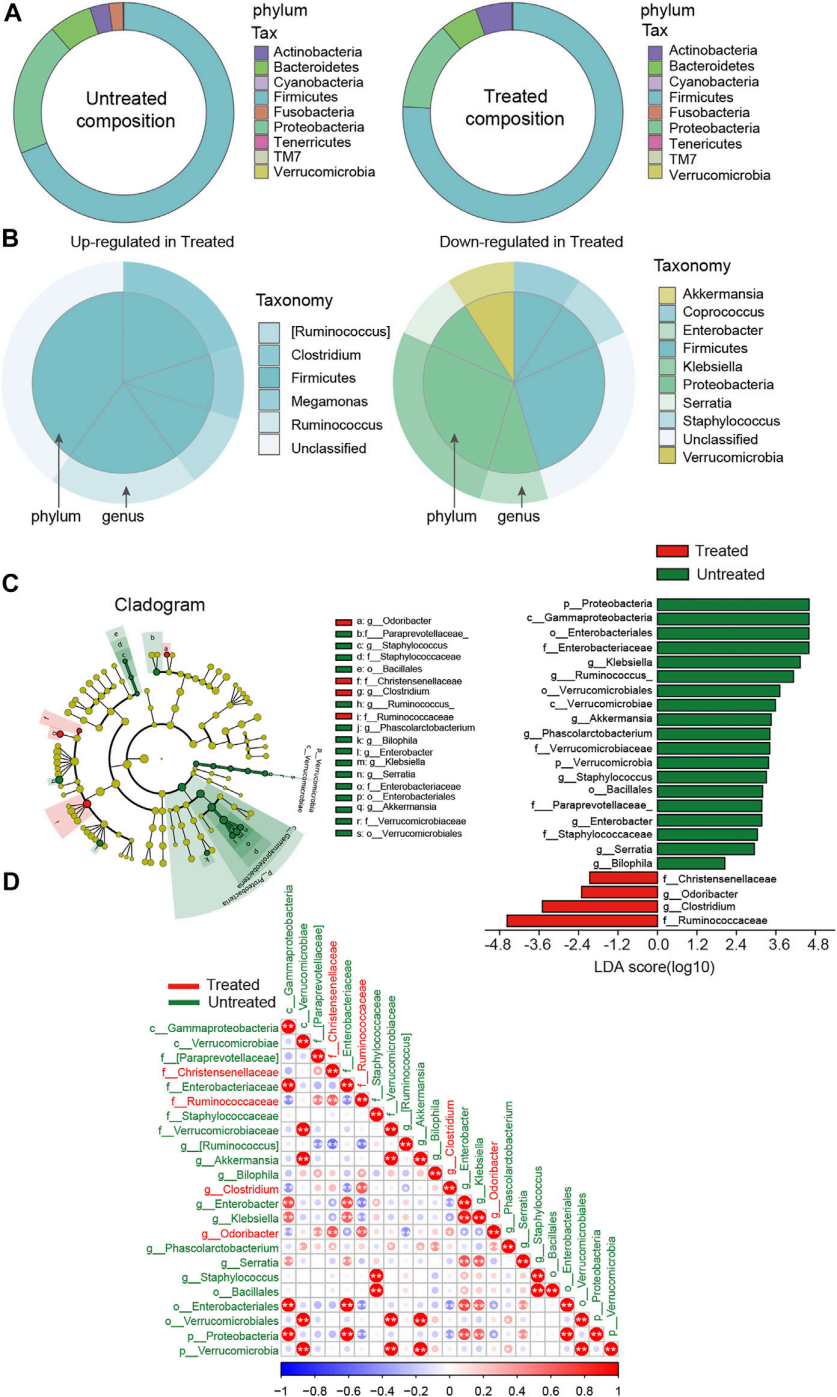
Disparity in gut microbial composition in the stool samples between untreated and ACEI/ARB-treated HTN patients. **(A)** Pie graph showing the bacterial proportion and composition of the overall nine phylum in untreated and treated groups. **(B)** As compared with the untreated group, the upregulated and downregulated genera and the corresponding phylum in the ACEI/ARB-treated group are shown in a pie chart, respectively. The outer circle represents a genus while the inner circle indicates the phylum it derives from. **(C)** LEfSe cladogram and LDA score analysis revealed differences in taxonomic composition, and the bacterial taxa significantly enriched the treated (green) and untreated (red) groups. **(D)** Heat map describing the correlation between different taxonomic compositions. A negative correlation is shown in red and a positive correlation in blue. The bacteria enriched in the treated patients are labeled with red color, while those dominant in the untreated patients are labeled with green. *, *p* < 0.05; **, *p* < 0.01, Spearman’s correlation.

To further identify the driving force for the observed alterations in dysbiosis of microbial communities among groups, differences in taxonomical composition between the groups were determined. A comparison was preliminarily performed between the treated and untreated groups. Firmicutes were predominant in both groups, with a higher relative abundance in the treated patients (Figure 2A). By contrast, the proportion of Proteobacteria was lower in the treated group. Moreover, Ruminococcus, Clostridium, and Megamonas*,* which belong to Firmicutes, were predominant in the treated group, while Coprococcus and Staphylococcus, Enterobacter and Klebsiella, along with Akkermansia were lower compared to the untreated group (Figure 2B). Using LEfSe analyses, the discriminant taxa between the untreated and treated groups were represented (Figure 2C). *Odoribacter* and *Clostridium* were clearly increased, while *Staphylococcus*, *Enterobacter*, and *Klebsiella* were drastically decreased in the treated patients. Figure 2D shows the heat map of the bacterial correlation in the treated patients and the untreated controls.

**FIGURE 3:**
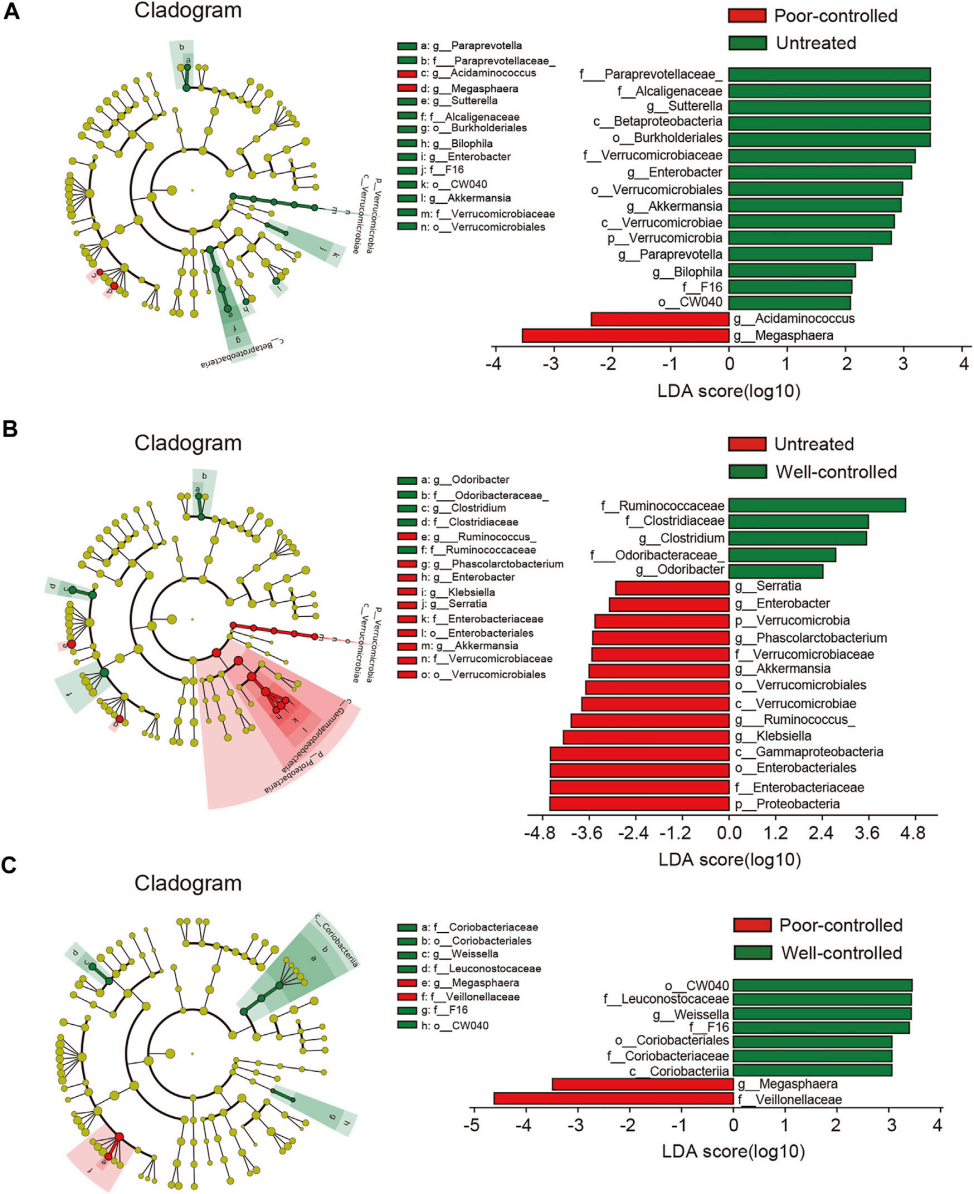
LEfSe analysis revealed differences in the fecal taxonomic composition between the poor-controlled or well-controlled hypertensive patients under ACEI/ARB therapy compared with untreated subjects. **(A)** Cladogram and LDA score of significantly different taxonomic compositions in poor-controlled (red) HTN patients relative to untreated (green) ones. **(B)** Significantly altered gut microbiota by ACEI/ARB in BP well-controlled individuals as compared with an untreated group are shown in LEfSe cladogram. Taxon abundant in untreated (red) and well-controlled (green) HTN patients are further presented with LDA scores. **(C)** Cladogram describing the microbial shifts in taxonomic composition between poor-controlled (red) and well-controlled (green) groups. The significance of different variables is defined by LDA scores (log10) > 2. Variants with *p* values < 0.1 are shown.

By subdividing patients into the well-controlled and poor-controlled groups, we further identified differences in relative abundance changes based on therapeutic effects. An LEfSe analysis demonstrated that 17 biomarkers were significantly different between the untreated and poor-controlled groups (Figure 3A). Enterobacter and Paraprevotella were lower in the poor-controlled group. Enterobacter and Paraprevotella have been previously confirmed to be extremely elevated in hypertensive patients or animals (Chang et al., 2020; Gutierrez-Calabres et al., 2020). In the well-controlled groups, the LDA scores revealed 19 biomarkers that were significantly different from the other groups (Figure 3B). Five bacterial taxa, including Clostridium, Odoribacter, Ruminococcaceae, Clostridiaceae, and Odoribacteraceae were clearly enriched in the well-controlled group. When examining the distinctions within the pharmaceuticals treated group, between well-controlled and poor-controlled groups, we found a spot of difference (Figure 3C). These gut bacteria altered among groups and showed apparent co-occurrence correlation with each other, indicating that the global shifts by ACEI/ARB treatment were collaborative (Supplementary Figure S5).

In addition, taxa discrimination between untreated and poor-controlled patients were regarded as not correlated with BP regulation due to indiscriminatingly high BP in both the groups. Likewise, the most important distinction between poor and well-controlled HTN groups was believed to be not ascribed to ACEI/ARB consumption. On the contrary, intestinal bacteria between well-controlled and untreated patients were indicated to be possibly linked to BP improvement under ACEI/ARB therapy. By excluding taxa that were neither associated with BP nor medical therapy, we obtained 11 shared taxa with those related to drug usage (untreated vs. treated) (Supplementary Figure S6). Proteobacteria, Gammaproteobacteria, Enterobacteriales, Enterobacteriaceae, Serratia, Phascolarctobacterium, Ruminococcus, and Klebsiella were progressively reduced from untreated to poor-controlled groups and ultimately to the well-controlled group, while Ruminococcaceae, Clostridium, and Odoribacter were found increased in well-controlled patients when compared to the untreated group.

### Microbial Functional Alterations in Hypertensive Patients Following Treatment

**FIGURE 4:**
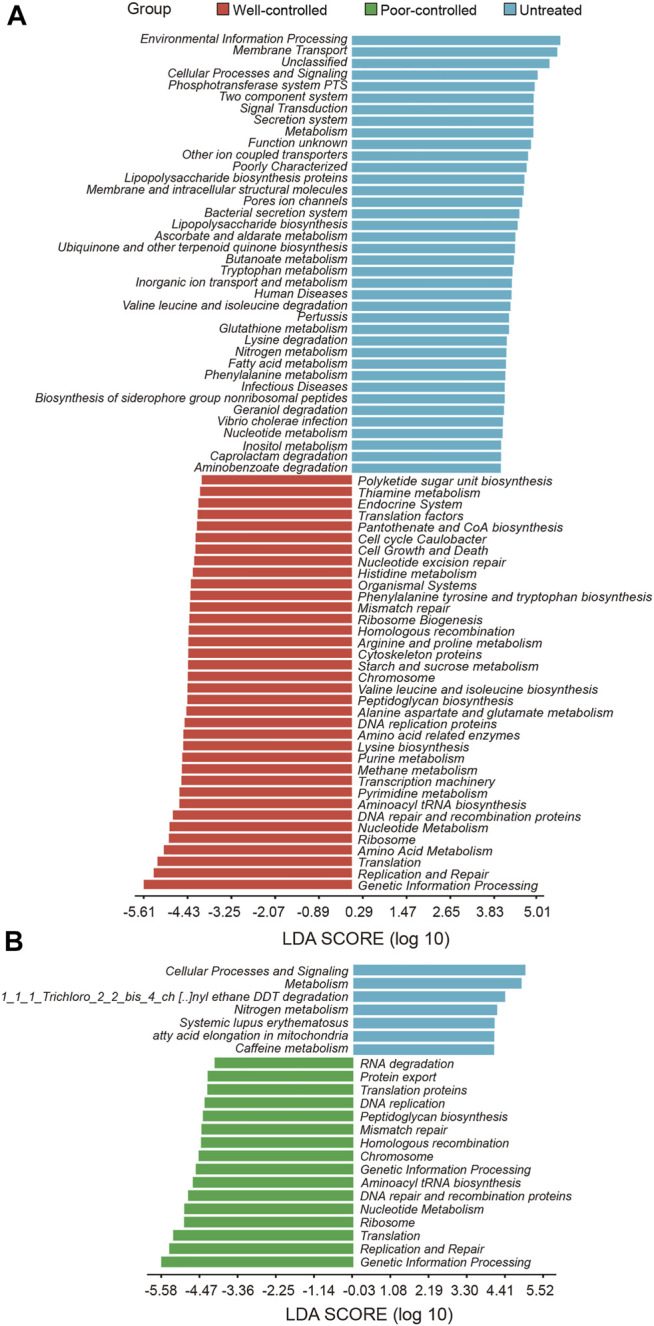
Potential functions of intestinal microbial metabolites in hypertensive individuals receiving ACEI/ARB or not receiving ACEI/ARB. **(A)** PICRUSt bar plots showing KEGG pathways significantly shifted in the well-controlled group (red) as compared with untreated patients (blue). Microbial functions targeting inositol metabolism were abundant in untreated patients and decreased in the ACEI/ARB well-control group. **(B)** Bar plot illustrating the KEGG pathways significantly distinct between untreated (blue) and poor-controlled group (green). The significance of different pathways is presented according to the LDA score. These variables with LDA scores (log10) > 2 are listed.

The potentials of metabolic and functional pathways in the gut microbiome of hypertensive patients were examined and analyzed through PICRUSt. We compared the KEGG pathways at level 3 and identified 74 KEGG categories with clearly differential abundance between the untreated and well-controlled groups **(** Figure 4A). The KEGG categories, including the bacterial secretion system, lipopolysaccharide biosynthesis, tryptophan metabolism, fatty acid metabolism, and phenylalanine metabolism inositol metabolism were prominently decreased in well-controlled patients. Comparisons between the untreated and poor-controlled groups for each KEGG functional category were further performed and are shown by the LDA score in Figure 4B. Most of the aforementioned potential microbial capacities suppressed in well-controlled patients were not found to be significantly improved in the poor-controlled groups. The terracomunal of well-controlled and poor-controlled groups mainly concentrated on promoting potential functions in peptidoglycan biosynthesis, replication and repair, nucleotide metabolism DNA repair, and recombination proteins.

### Patients Subjected to Medicine Display Dramatic Changes in Serum Metabolome

**FIGURE 5:**
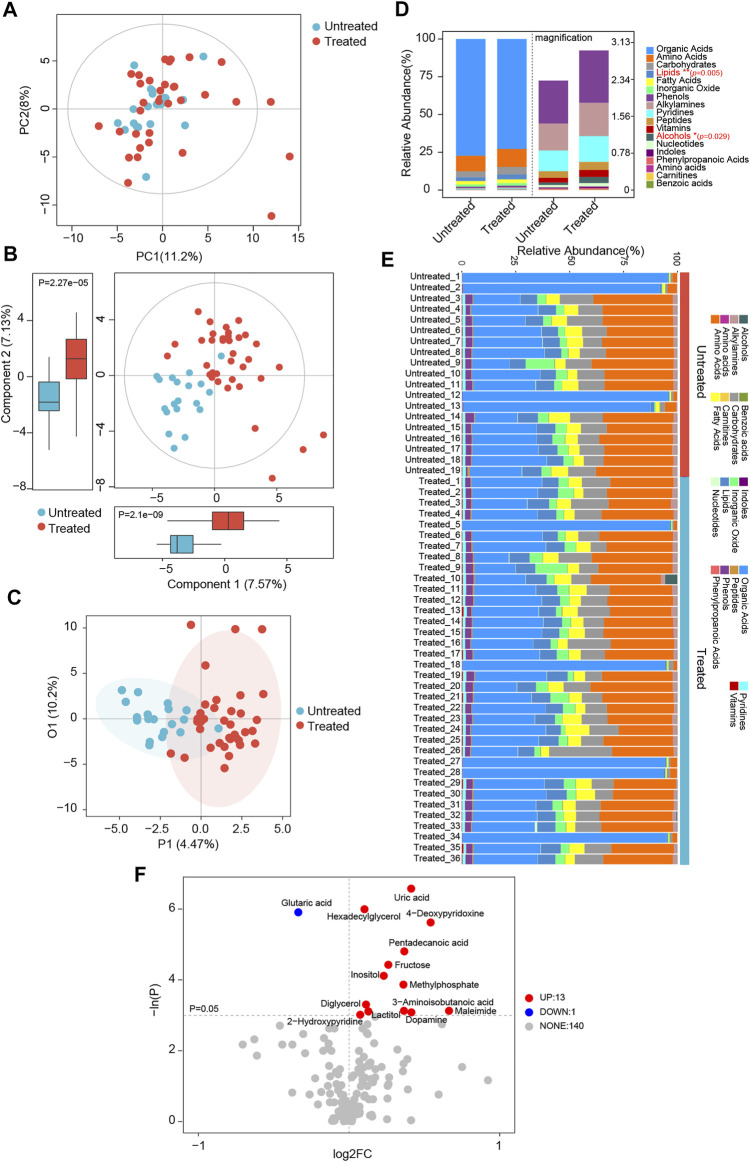
Global patterns for the serum metabolome in ACEI/ARB-treated HTN patients. **(A)** PCA plots depicting the metabolic profiles and features according to untargeted GC-TOF/MS data of serum samples from untreated and treated hypertensive groups. PC1, principal component 1; PC2, principal component 2. **(B)** Scatter plots of PLS-DA identifying the metabolic discrimination and separation between groups. *p* = 2.1e−09 and 2.27e−05 between untreated and treated groups were obtained at Component 1 and Component 2, respectively. **(C)** OPLS-DA with scatter plots shows the metabolic discrimination and separation between groups. **(D)** Relative abundance and percentage of serum metabolites and class composition detected in untreated and treated HTN patients. *p < 0.05, **p < 0.01. **(E)** Relative abundance of annotated metabolite class in serum samples of each individual from untreated and treated groups. **(F)** Volcano plots show significant alterations of serum metabolites between groups. Metabolites at *p* < 0.05 were considered to be significantly distinct. Up, the number of significantly elevated serum metabolites in the treated group as compared with untreated HTN patients; Down, the number of dramatically decreased metabolites in treated group; None, the number of metabolites not significantly altered between groups. Each metabolite is labeled with the corresponding annotation.

Based on the serum metabolomics data, discrimination between the patients with and without treatment was assessed using PCA, PLS-DA, and OPLS-DA clustering analyses. As observed in the PCA plots, there was an overlap of the samples derived from distinct groups; treated patients could not be clearly distinguished from untreated ones (Figure 5A). Nonetheless, according to serum metabolic profiles, the PLS-DA scatter plots showed obvious separation between the treated and untreated groups (Figure 5B). In components 1 and 2, the patients under ACEI/ARB therapy showed significantly different scores than the untreated controls (Figure 5B). On the other hand, individuals in the treated group were distinguished from the others, as evidenced by the OPLS-DA score plots (Figure 5C). For a detailed metabolic composition, all the metabolites identified were classified into 18 classes, and the proportion of each is described in Figures 5D,E. The serum samples of individuals in the treated or untreated groups mainly consisted of organic acids, amino acids, carbohydrates, lipids, and fatty acids. Compared with the untreated ones, patients following medication had significantly higher levels of lipids and alcohol. Among all the 154 metabolites detected, the differentially abundant metabolites in the serum of patients were explored. Notably, 14 metabolites were observed to be discriminatory between the groups (Figure 5F). Interestingly, in treated patients, almost all the significantly altered metabolites were elevated.

Similarly, when further comparison was performed between poor-controlled and untreated, well-controlled and untreated, and well-controlled and poor-controlled patients, respectively, the PCA plots again failed to completely distinguish the different groups (Supplementary Figures S7A, 8A, 9A). Consistent with the aforementioned observations, scatter plots of PLS-DA at both components 1 and 2 obviously discriminated patients from different groups (Supplementary Figures S7B, 8B, 9B). OPLS-DA score plots further verified the distinctions of metabolic features between the groups (Supplementary Figures S7C, 8C, 9C). Among the 18 metabolic classes, the abundance of fatty acids and alcohols was different between poor-controlled and untreated ones; lipids and vitamins were unequal between well-controlled and untreated ones; but none was identified to be prominently distinct between well-controlled and poor-controlled patients (Supplementary Figures S7D, 8D, 9D).

**FIGURE 6:**
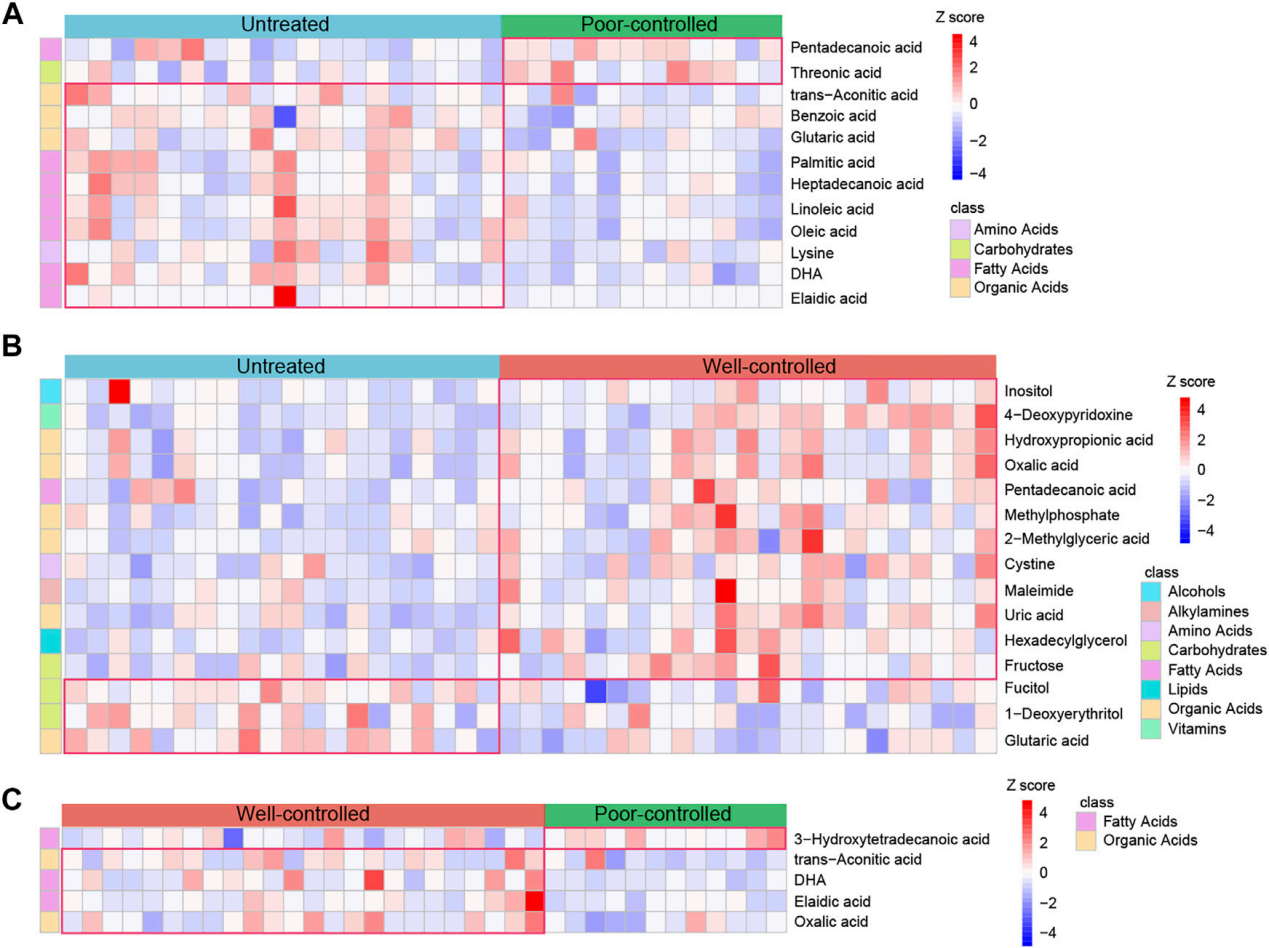
Abundance of the serum metabolites in untreated, ACEI/ARB well-controlled, and ACEI/ARB poor-controlled groups. **(A)** Heat map for the relative abundance of strikingly decreased and significantly enriched serum metabolites in poor-controlled patients when compared with untreated ones. The metabolites shown are statistically significant in the calculation, and the relative abundance is shown in each sample. **(B)** Heat map illustrating the relative abundance of shifted serum metabolites between untreated and ACEI/ARB well-controlled groups. **(C)** Heat map demonstrating the relative abundance of statistically shifted distinct serum metabolites between the poor-controlled and well-controlled groups. The abundance profiles are transformed into Z-scores by subtracting the average abundance and dividing the standard deviation of all the samples. Z-score is negative and shown in blue when the abundance is lower than the mean and is positive and shown in red when the relative abundance is higher than the mean.

Compared to untreated patients, there were 10 reduced metabolites and 2 enhanced in poor-controlled individuals, whereas 3 metabolites were reduced and 12 were elevated in well-controlled ones (Supplementary Figures S7E, 8E). However, five different metabolites were detected between patients of the well-controlled and poor-controlled groups by therapy (Supplementary Figure S9E). The relative abundance of these discriminatory metabolites between the groups is described in Figure 6. Fatty acids such as elaidic acid, oleic acid, linoleic acid, and palmitic acid were depleted in the poor-controlled group, whereas alcohols inositol, organic acids hydroxy propionic acid, oxalic acid, and fatty acid pentadecanoic acid were augmented in the well-controlled group, as compared with HTN patients with no history of treatment (Figures 6A,B). Oxalic acid was observed to be increased in well-controlled patients relative to both untreated and poor-controlled individuals (Figure 6C). The varied serum metabolites identified among groups were confirmed to be significantly correlated with fecal microbiota (Supplementary Figure S10). Inositol was found to be negatively correlated with opportunistic pathogens previously reported, such as Klebsiella, Enterobacter, and Proteobacteria.

**FIGURE 7:**
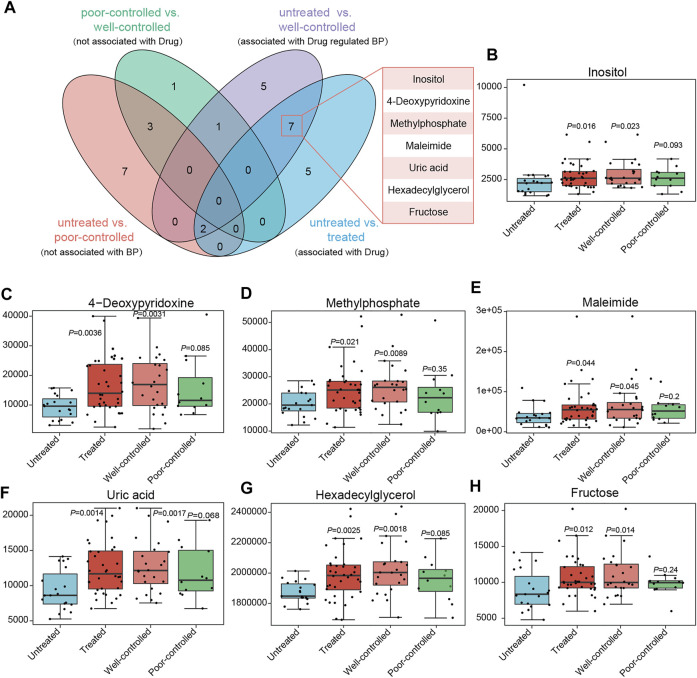
Core metabolites significantly altered between groups, possessing potential association with medical treatment or BP regulation. **(A)** Venn diagrams indicate the metabolites in different groups. The variables different between the untreated and poor-controlled groups are considered as not associated with BP attenuation; those between the poor-controlled and well-controlled groups are not attributed to ACEI/ARB treatment; variables derived from untreated vs. treated might be linked to drug therapy; when comparing the untreated and well-controlled groups, it is intended to obtain serum metabolites related to BP regulation by ACEI/ARB administration. Potential metabolites important for ACEI/ARB in lowering BP are concentrated in 7. **(B–H)** Relative abundance of inositol, 4-deoxypyridoxine, methylphosphate, maleimide, uric acid, hexadecylglycerol, and fructose in ACEI/ARB-treated HTN patients either well-controlled or poor-controlled.

Additionally, metabolites those altered between untreated versus poorly controlled hypertensive patients were not associated with BP regulation due to indiscriminate BP levels in these individuals. As both poor and well-controlled HTN patients received medical treatments, the other factors were speculated to be accountable for their distinctions. Among the metabolites found to be different in well-controlled versus untreated patients, which were highly possible to be linked with BP modulation under ACEI/ARB therapy, we excluded those neither associated with BP nor with medical therapy, and we further obtained seven shared ones with untreated versus treated patients (related to drug usage) (Figure 7A). Inositol, 4-deoxypyridoxine, methylphosphate, maleimide, uric acid, hexadecylglycerol, and fructose showed progressively increased in abundance from untreated to poor-controlled groups and finally to the well-controlled group, with marginal statistical significance in poorly controlled patients and more robust statistical difference in well-controlled versus untreated patients (Figures 7B–H).

## Discussion

This study found no obvious difference in gut microbial richness in terms of alpha diversity or microbial structures according to the beta diversity in patients with essential HTN receiving ACEI/ARB therapy when compared with untreated ones. However, when further examining taxa, potential functions, and serum metabolome, HTN patients who had received ACEI/ARB treatment showed significant alterations, especially those in the well-controlled group. Some potentially pathogenic bacteria such as Enterobacter and Klebsiella were reduced, whereas several beneficial bacteria, e.g., Odoribacter increased after ACEI/ARB therapy. Furthermore, some favorable metabolites, such as pentadecanoic acid and inositol, increased in the well-controlled group as compared with untreated patients. Moreover, a correlation analysis suggested that modulations of the intestinal microbial composition and functions exerted by anti-hypertensive treatment were directly linked to changes in metabolites and might ultimately lead to the improvement of the host BP.

Over the recent years, many studies have been focused on examining the correlation between gut microbial ecosystems and non-antibiotic drugs (Vich Vila et al., 2020). Stomach acid inhibitors of proton pump inhibitors have been reported to be associated with the enrichment of the typical oral microbes colonized in the gut (Imhann et al., 2016; Jackson et al., 2016). Statin, a first-line agent for dyslipidemia and atherosclerosis, can modulate the gut microbiome of acute coronary syndrome patients toward a healthier status by interacting with metabolites, such as fatty acids and prenol lipids (Hu et al., 2021). Meanwhile, the effect of some anti-hypertensive drugs on the intestinal flora has also been investigated. Evidence obtained from animal experiments showed that candesartan could normalize the ratio of Firmicutes to Bacteroidetes under hypertensive status and further enhance the amount of fecal short-chain fatty acids (Wu et al., 2019). A recent study examining residents in Henan Province suggested that the abundance of gut Megasphaera and Megamonas was positively correlated to SBP in hypertensive patients receiving anti-hypertensive treatment (Li et al., 2019). Another study found that *Bacteroides fragilis* and *Klebsiella pneumoniae* were increased, while *Dorea longicatena* and *Clostridium leptum* were reduced in the gastrointestinal tract of HTN patients consuming anti-hypertensive medications compared to normotensive healthy controls (Zheng et al., 2021). Nevertheless, the main limitation was that they primarily concentrated on HTN patients, failing to take the anti-hypertensive treatment into account or the impacts of anti-hypertensive drugs. In this study, we evaluated the effects of ACEI/ARB on the gut microbiome and metabolites in untreated, poor-controlled, and well-controlled HTN patients according to the anti-hypertensive efficacy.

Preliminarily, a comparison was conducted between the untreated and treated HTN patients. Odoribacter and Clostridium were clearly increased, and Proteobacteria, Enterobacter, and Klebsiella, were decreased in the treated patients. A previous study reported that HTN is associated with an elevated Firmicutes/Bacteroidetes ratio, overrepresented opportunistic pathogenic bacteria (e.g., Proteobacteria, Enterobacteriaceae, *Enterobacter*, and *Klebsiella*), and depleted populations of acetate-/butyrate-producing taxa such as Odoribacter (Mell et al., 2015; Gomez-Arango et al., 2016; Li et al., 2017; Yan et al., 2017; Jin et al., 2021). Klebsiella, a pathogenic bacteria frequently detected in the human gastrointestinal tract, has been strongly associated with pneumonia, inflammatory bowel diseases, and several other diseases (Dorman and Short, 2017; Lin et al., 2020).Moreover, excessive growth of Klebsiella has been considered as a precursor of intestinal microbiota disorders (Yan et al., 2017), leading to a variety of serious chronic diseases, such as colitis (Garrett et al., 2010), Crohn’s disease (CD), and ankylosing spondylitis (Ebringer et al., 2007). In the present study, we found bacteria such as Klebsiella were predominant in untreated hypertensive patients, while Clostridium was dominant in the ACEI/ARB group, especially in well-controlled individuals. According to our observations in a previous investigation, which aimed at exploring the discrepancy between healthy controls and untreated hypertensive individuals, the dominated intestinal microbiota identified in healthy controls (*vs.* hypertensive patients) included Clostridium, Faecalibacterium, and Blautia, and the scarce bacteria consisted of *Klebsiella* and *Prevotella* (Li et al., 2017). The enrichment of Clostridium and deficiency of Klebsiella previously reported in healthy controls are similar to the current findings in the ACEI/ARB treated group.

The facultative anaerobic Gram-negative bacilli, Enterobacter, is a common cause for nosocomial infections of the bloodstream and urinary tract, as well as meningitis (Liu et al., 2013; Pendleton et al., 2013). Odoribacter, an anaerobic non–spore-forming, nonmotile, Gram-negative bacterium, was confirmed to ferment carbohydrates and produce protective acetic, propionic, succinic, butyric, isovaleric, and isobutyric acids *in vitro* (Nagai et al., 2010; Göker et al., 2011). A reduced abundance of Odoribacter has been identified in the fecal microbiome of CD patients, which was characterized by scarce butyrate-producing capacity (Morgan et al., 2012). Most importantly, the enrichment of Odoribacter within the gut microbiota was indicated to be inversely correlated with host BP levels (Gomez-Arango et al., 2016). In this study, we found that ACEI/ARB treatment significantly reduced gut Enterobacter and Klebsiella, but enhanced Odoribacter in HTN patients. Similar results were detected in the BP of well-controlled HTN patients under therapy versus the untreated patients. Moreover, we also found that Odoribacter was negatively correlated with Enterobacter and Klebsiella. Overall, we speculated that ACEI/ARB therapy might modulate the intestinal flora of HTN patients by improving it toward a healthy state, at least to some extent.

Prediction for gut microbial community’s functional capabilities indicated that gut microbiome may elicit impacts on host physiology and pathology through interference of metabolic functions. Compared with the untreated patients, our present study further demonstrated that the potential functions of the gut microbiota were dramatically shifted in the individuals with well-controlled BP under treatment. Several metabolic pathways involved in amino acids biosynthesis and transport, including lysine biosynthesis and isoleucine biosynthesis, were reported to be deficient in hypertensive patients (Li et al., 2017) and were significantly promoted in ACEI/ARB treated patients manifesting well-controlled BP. Correspondingly, certain microbial pathways related to the degradation of these amino acids (e.g., lysine, isoleucine) were identified to be suppressed in the well-controlled group. In addition, the therapy-improved KEGG pathways (phosphotransferase system, biosynthesis of lipopolysaccharide, and secretion system) were dominant in HTN naive patients. Although the functional annotation analyses of the gut microbiome are predictive, they provide certain clues that modulated intestinal flora by ACEI/ARB treatments, which might facilitate a health-related performance by ameliorating physiological and metabolic functions.

Next, we examined the gut microbe–host metabolism interactions under ACEI/ARB therapy. As a wide range of biologically active end products produced by the gut microbiota could be released into the peripheral circulation and thus exert important interactions with multiple organs of the host, we explored the serum metabolomics changes among untreated, poor-controlled, and well-controlled HTN patients. Interestingly, the metabolic features (PLS-DA and OPLS-DA plots) were different between the groups. Compared with HTN naive patients, almost all the significantly altered metabolites were decreased in treated HTN patients, e.g., pentadecanoic acid and inositol. The enrichment of pentadecanoic acid and inositol was further confirmed in poor-controlled and well-controlled HTN patients compared with untreated HTN patients. Pentadecanoic acid is a dairy-specific odd-chain fatty acid that can suppress inflammation and oxidative stress (Wang et al., 2011; Weitkunat et al., 2017). Previous studies have indicated that pentadecanoic acid is inversely associated with total cholesterol and low-density lipoprotein cholesterol levels (Samuelson et al., 2001; Warensjö et al., 2010; Wu et al., 2020) and is further linked to a lower incidence of type 2 diabetes mellitus (Kröger et al., 2011; Santaren et al., 2014). For the nutrients in the family of simple carbohydrates, inositol is commonly found in daily food, especially fresh fruits and vegetables, legumes, and cereals (Hashemi Tari et al., 2021). Accumulating evidence has indicated that inositol has an essential role in insulin signaling and vascular endothelial function improvement, which is regarded as adjunctive therapy for various metabolic diseases, such as insulin resistance and HTN (Giordano et al., 2011; Donà et al., 2012). In addition, animal experiments have demonstrated that inositol supplementation could enhance the activity of inositol receptors and modulate vascular and BP levels *via* inositol 1,4,5-triphosphate receptor–mediated Ca^2+^ release (Lin et al., 2019; Buckley et al., 2020). Consistently, inositol has been frequently shown to be beneficial for lowering SBP and DBP (Kim et al., 2005; Costantino et al., 2009; Giordano et al., 2011). In this study, serum inositol was negatively correlated with HTN-related opportunistic pathogens, such as Klebsiella, Enterobacter, and Proteobacteria. Based on the aforementioned observations, we speculate that ACEI/ARB therapy might improve the growth of specific gut bacteria in HTN patients, which participate in the production and metabolism of certain biologically active metabolites, thus lowering the host BP. Yet, this inference needs to be further investigated and validated.

Indeed, it has been indicated that the bacterial members in the gut might modulate the availability and efficacy of therapeutic drugs (Klünemann et al., 2021). Most recently, investigators have confirmed that the gut microbiome–encoded enzymes to selectively inactivate acarbose, a clinically used antidiabetic drug (Balaich et al., 2021). Therefore, the intestinal bacteria and metabolites might also affect the pharmacodynamics of the ACEI/ARB drugs, leading to a discrepancy in the treatment outcome of the well-control and poor-control groups. By comparison between well-controlled and poor-controlled patients, it was identified that Leuconostocaceae, Coriobacteriales, and Weissella were enriched in well-controlled patients, while Veillonellaceae and Megasphaera were dominant in patients with poor-controlled BP. Additionally, five metabolites were identified to be different between patients of well-controlled and poor-controlled groups by therapy, with trans-aconitic acid, DHA, elaidic acid, and oxalic acid increased in the well-controlled group, and 3-hydroxytetradecanoic acid in the poor-controlled group.

This study also has a few limitations. This is a cross-sectional study with a relatively small sample size, lower proportion of females, and the microbial and metabolic features evaluated at status quo instead of monitoring dynamic changes. In addition, although normotensive healthy controls were not included in this study, we found the SBP (128.84 mmHg, 95%CI: 121–134.58 mmHg) and DBP (78.5 mmHg, 95%CI: 72.67–84.67 mmHg) of the well-controlled group were a little higher than an ideal normotensive level. Further studies examining the microbial and metabolic profiles between the well-controlled group and healthy controls should be carried out in the future to uncover their discrepancy. Furthermore, information for confounding factors such as dietary habits, lifestyles, duration, and compliance of taking ACEI/ARB anti-hypertensive medications, which might lead to a bias in the analysis failed to be obtained. At last but not least, identifying gut microbiota by 16S rRNA sequencing might introduce biases through PCR amplification steps and acquire genus data to a maximum extent for identification. Further investigations with a larger sample size with balanced male and female distribution to validate and verify the findings in the present study are still necessary.

## Conclusion

Our data, which were based on multi-omics technology, indicated that ACEI/ARB therapy could modulate the gut microbiota and metabolites in HTN patients, showing a tendency toward a healthy status. Faced with the higher prevalence and lower control rate of HTN in China, our findings provide a new insight aiming at relieving HTN and advancing therapeutic drug effects by ameliorating dysbiosis and restoring homeostasis of the gut microbiota by improving dietary habits and lifestyles or supplementing with probiotics and prebiotics.

## Data Availability

The data sets presented in this study can be found in online repositories. The names of the repository/repositories and accession number(s) can be found in the link EMBL European Nucleotide Archive under BioProject accession code PRJEB50682 (http://www.ebi.ac.uk/ena/data/view/PRJEB50682).
