# Supplementary figures and images for: Antihypertensive Therapy by ACEI/ARB Is Associated With Intestinal Flora Alterations and Metabolomic Profiles in Hypertensive Patients
Source: Front Cell Dev Biol. 2022 Mar 23;10:861829. doi: 10.3389/fcell.2022.861829 (PMC8986158; doi:10.3389/fcell.2022.861829)

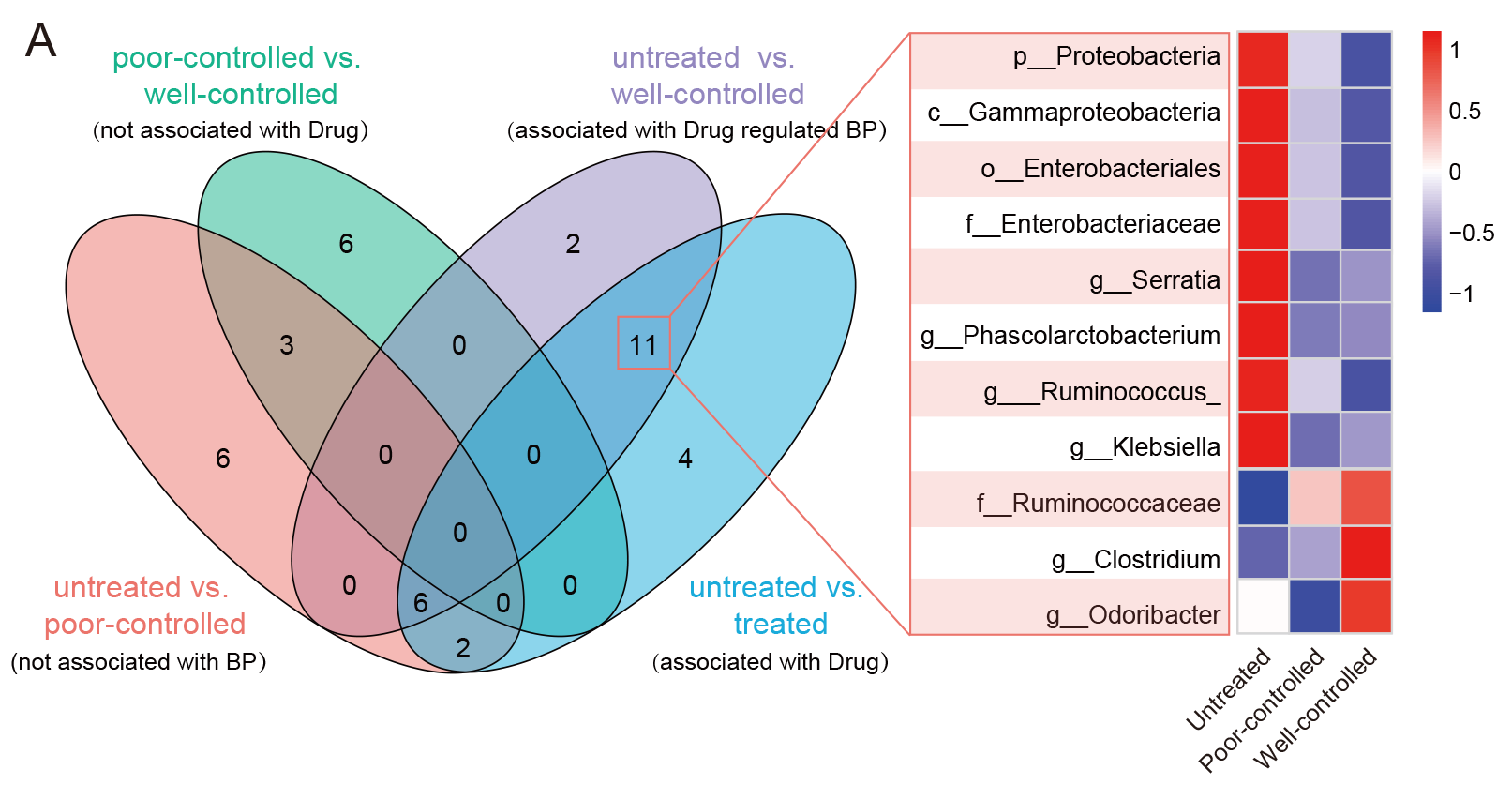

Supplement: Supplementary file 1 [file Image6.TIF]

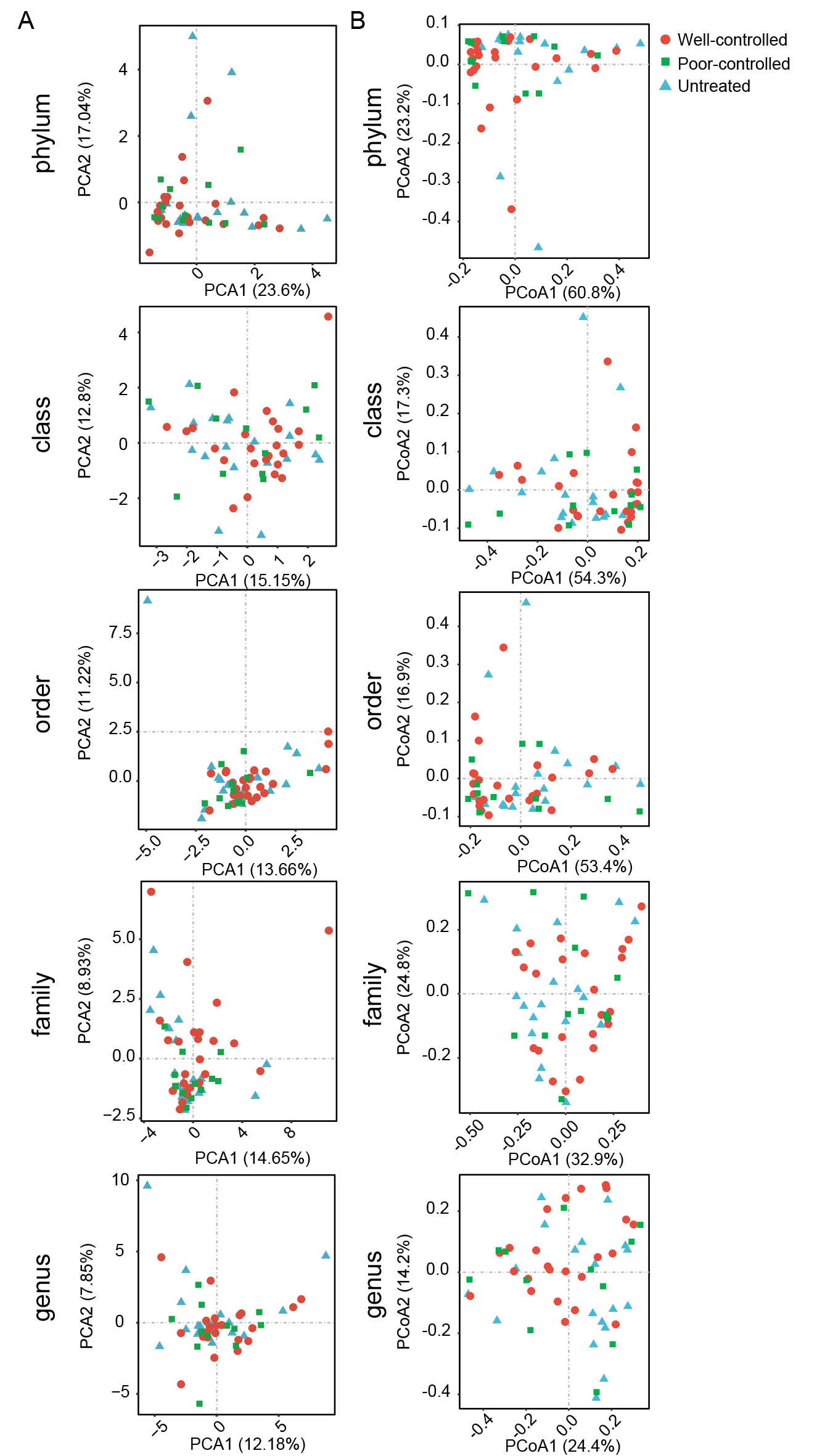

Supplement: Supplementary file 3 [file Image3.TIF]

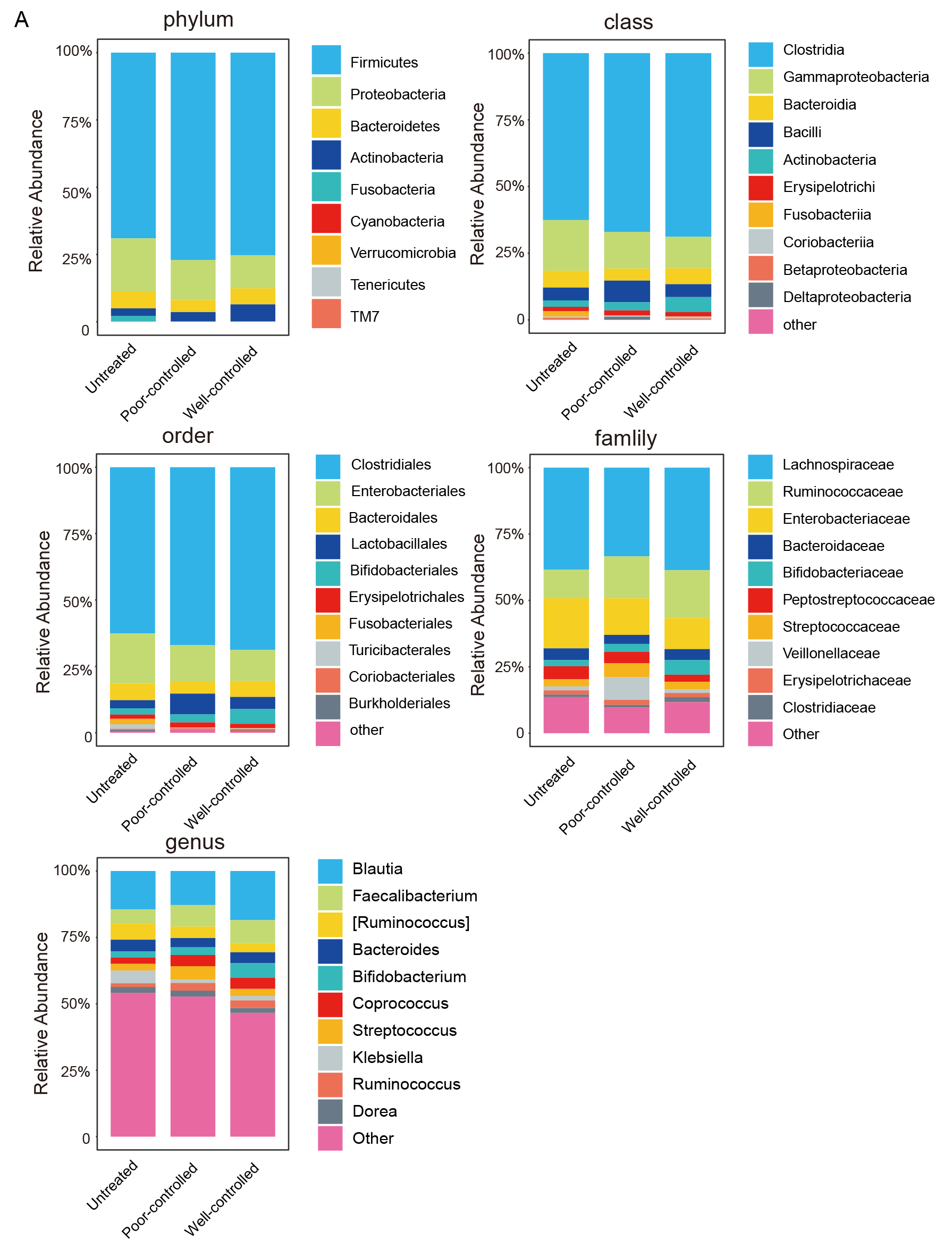

Supplement: Supplementary file 4 [file Image4.TIF]

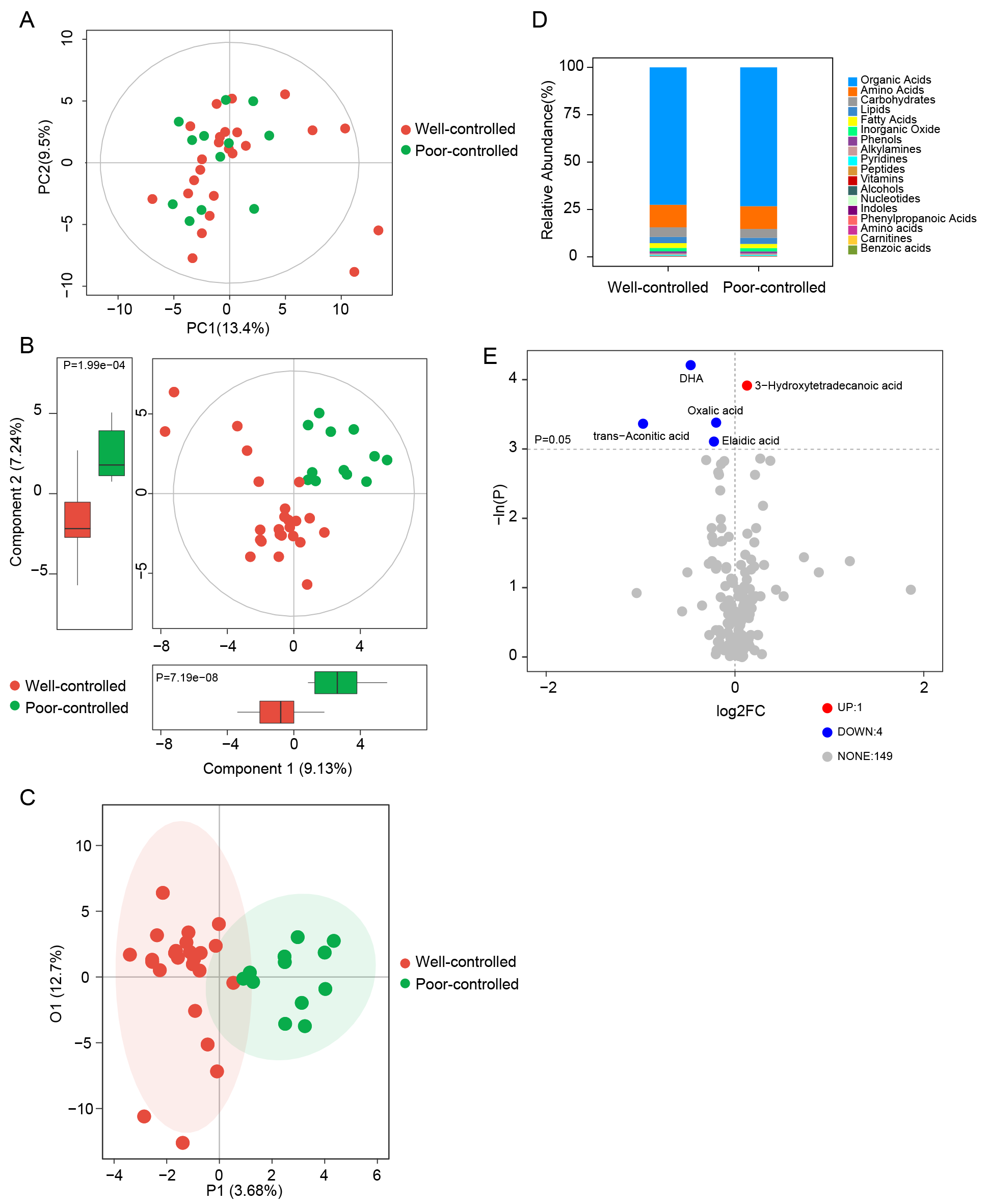

Supplement: Supplementary file 5 [file Image9.TIF]

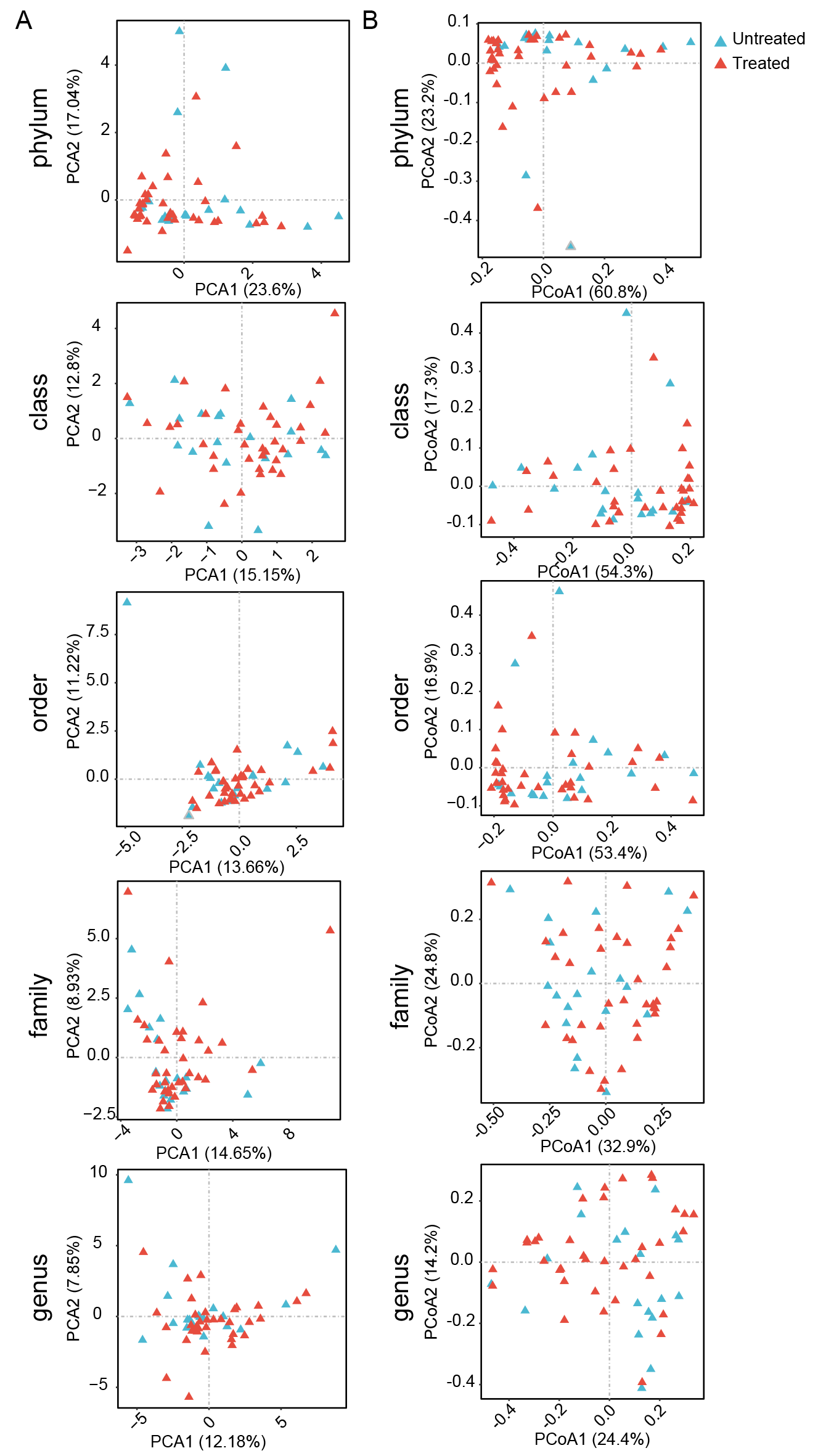

Supplement: Supplementary file 6 [file Image2.TIF]

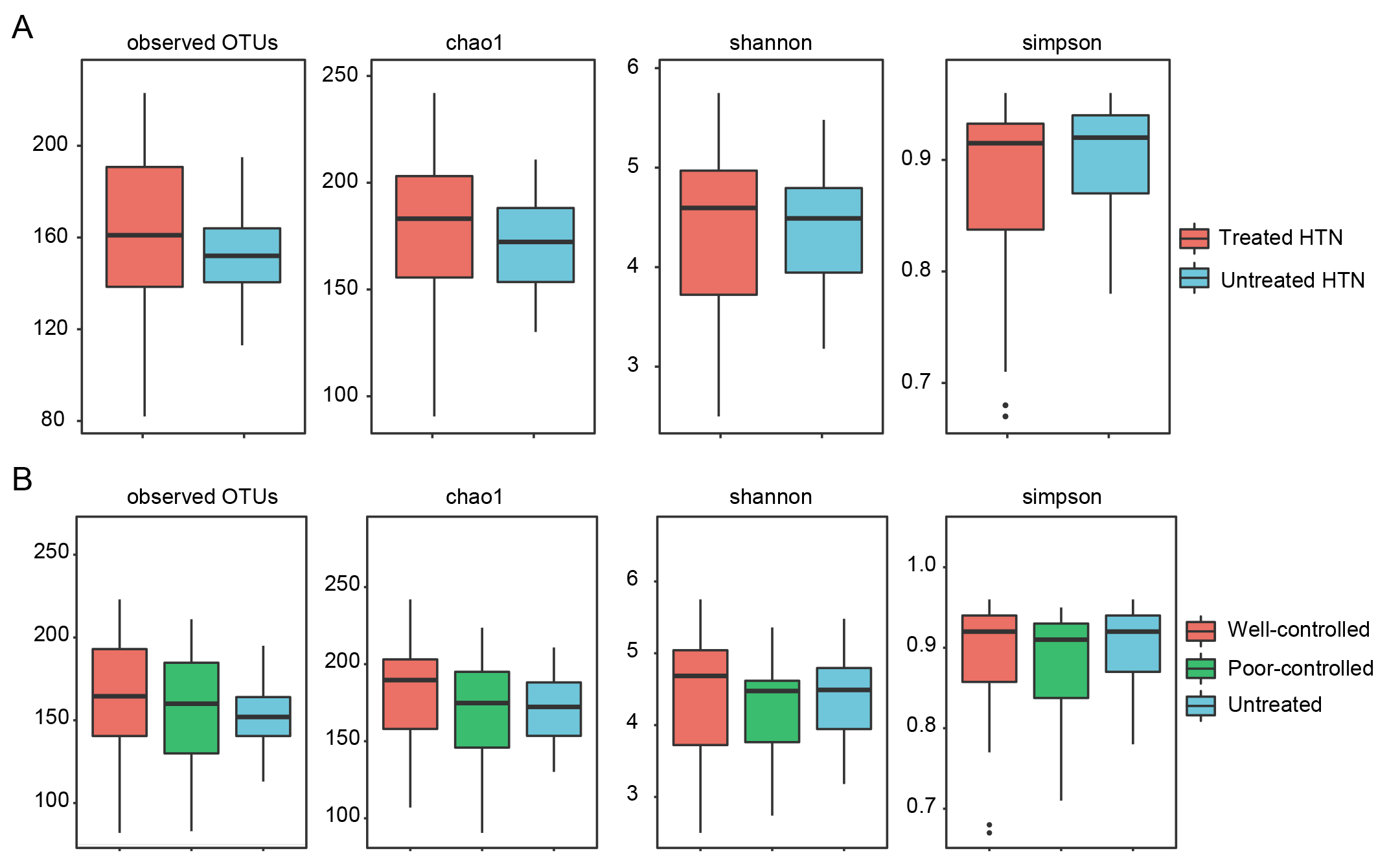

Supplement: Supplementary file 7 [file Image1.TIF]

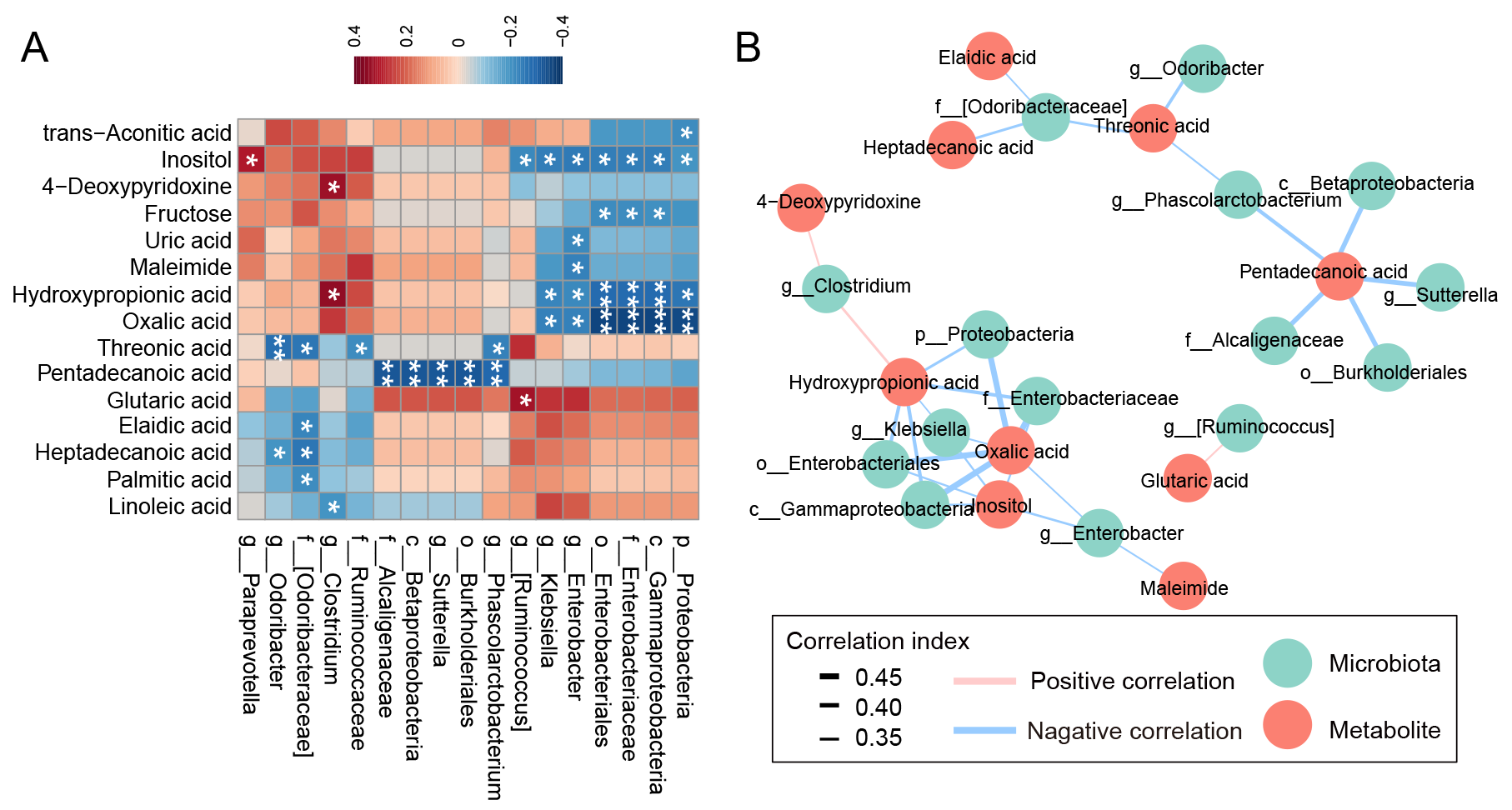

Supplement: Supplementary file 8 [file Image10.TIF]

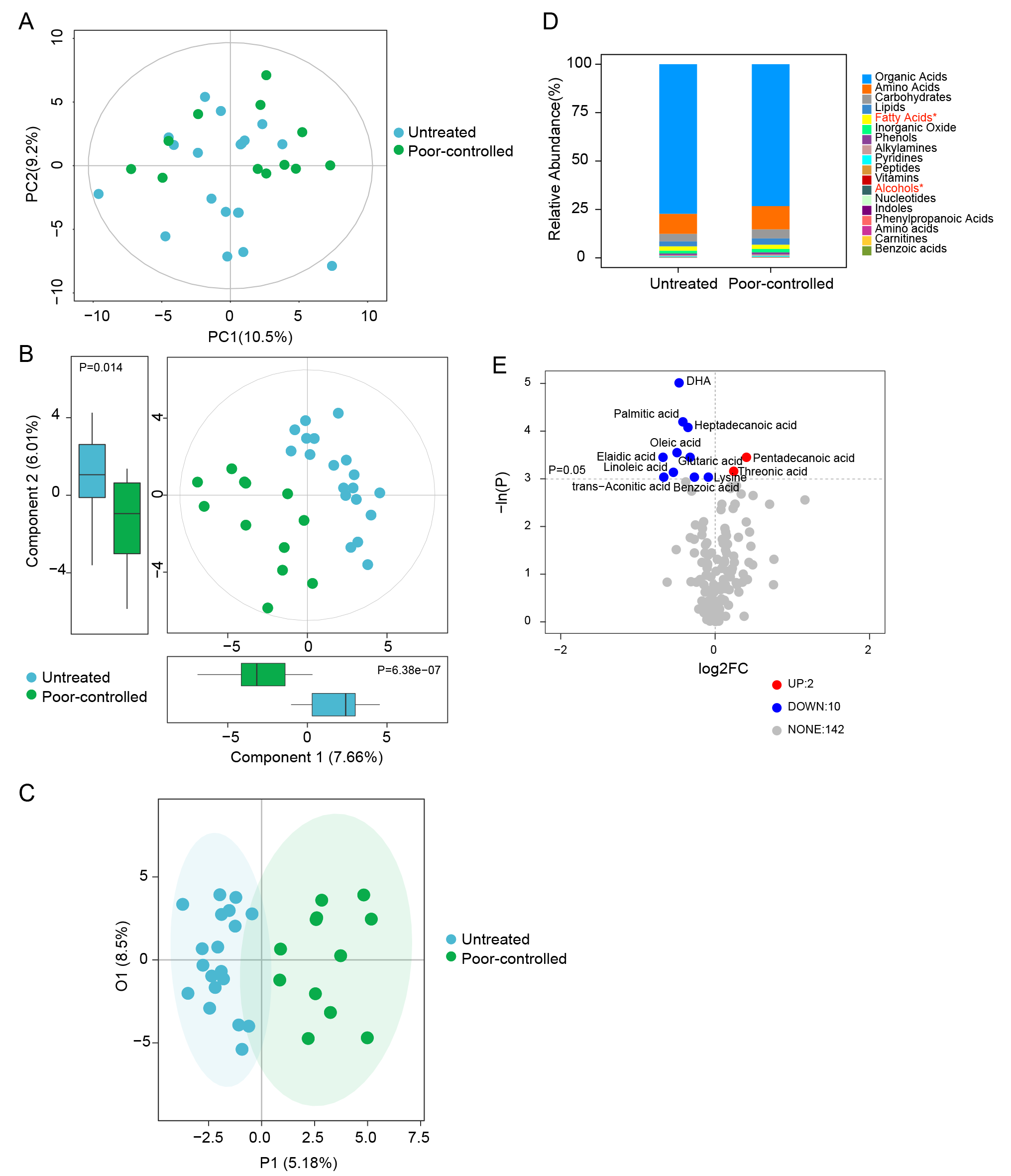

Supplement: Supplementary file 9 [file Image7.TIF]

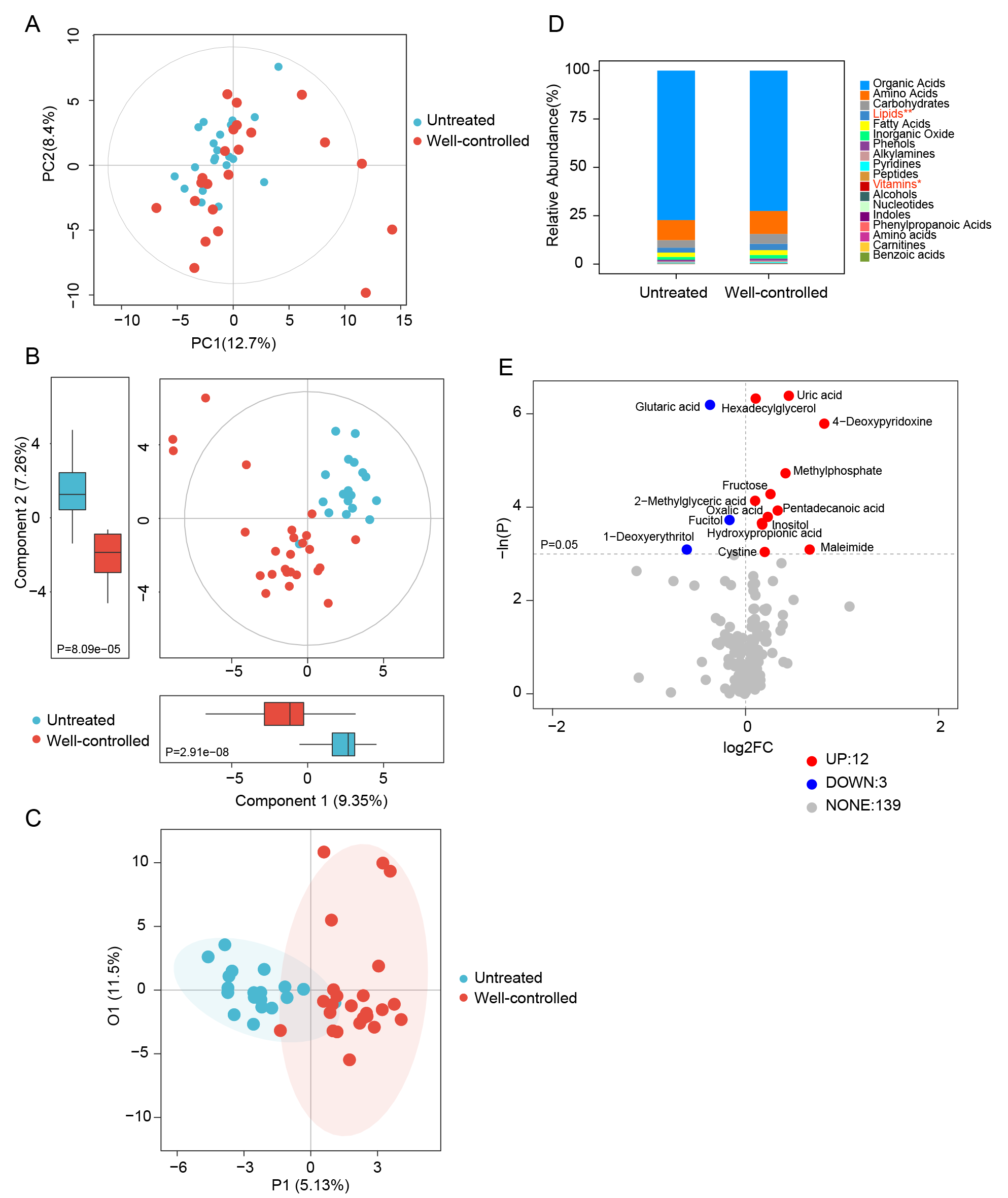

Supplement: Supplementary file 10 [file Image8.TIF]

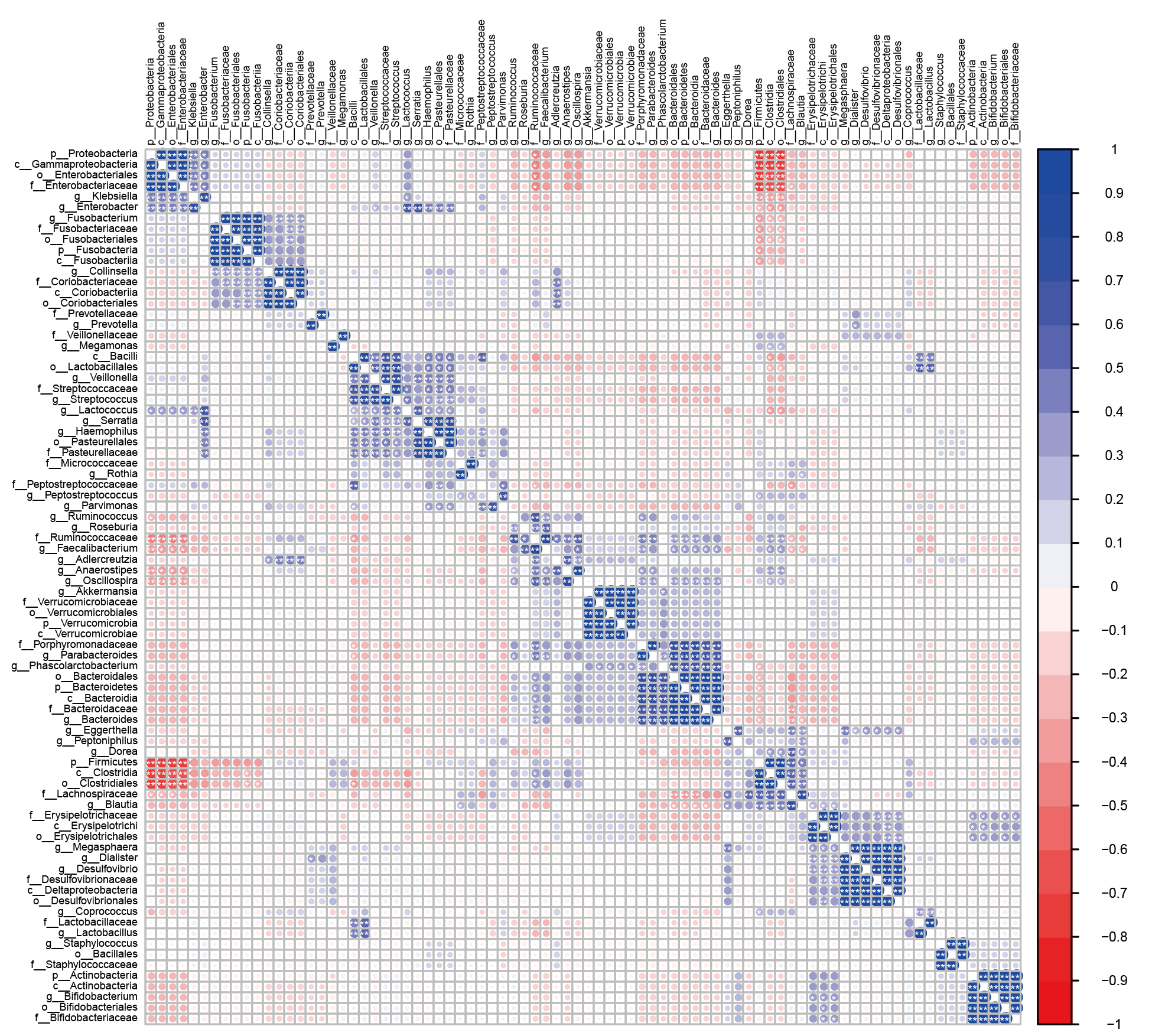

Supplement: Supplementary file 11 [file Image5.TIF]
