# Supplementary material for: Antihypertensive Therapy by ACEI/ARB Is Associated With Intestinal Flora Alterations and Metabolomic Profiles in Hypertensive Patients
Source: Front Cell Dev Biol. 2022 Mar 23;10:861829. doi: 10.3389/fcell.2022.861829 (PMC8986158; doi:10.3389/fcell.2022.861829)
Supplement: Supplementary file 2 [file Table1.docx]

Supplementary Material

# Supplementary Figures and Tables

**Supplementary Figure S1. Alpha diversity of intestinal microbiota in untreated and ARB/ACEI treated hypertensive patients based on the OTU profiles.** **(A)** The gut microbial richness and alpha-diversity in untreated (n=19) and treated (n=36) hypertensive patients accessed using OTU numbers, Chao1 richness, Shannon index, and Simpson index, respectively, according to the relative abundance of OTUs in each sample. **(B)** The observed OTUs and alpha diversity parameters of Chao1 richness, Shannon index, and Simpson index were further determined in ARB/ACEI treated HTN patients with BP well-controlled or poor-controlled. Well-controlled group, n=24; Poor-controlled group, n=12. The boxes represent the interquartile ranges, the inside line represents the median, and the circles represent outliers.

**Supplementary Figure S2. Beta diversity of gut flora between untreated and treated hypertensive patients. (A)** PCA plots describing distance among hypertensive individuals receiving ARB/ACEI treatment or not were performed based on the microbial abundance at phylum, class, order, family, and genus level, respectively. **(B)** Scatter plots of PCoA were constructed based on the abundance profiles of the gut microbiome in phylum, class, order, family, and genus, respectively. Triangles in blue indicate samples from the untreated group, and red indicate individuals from the treated group.

**Supplementary Figure S3. Beta diversity analysis of fecal microbiota in** **well-controlled or poor-controlled following ARB/ACEI therapy. (A)** PCA plots depicting the extent of separation among groups were based on the phylum, class, order, family, and genus, respectively. **(B)** PCoA plots of participants in groups were carried out based on the taxon annotation in phylum, class, order, family, and genus, respectively. Circles in red indicate samples from the well-controlled group; squares in green indicate individuals from the poor-controlled group; triangles in blue indicate samples from the untreated group.

**Supplementary Figure S4. Most enriched gut microbial taxon annotated in the stool samples among untreated, poor-controlled, and well-controlled groups. (A)** Bar plots showing the relative abundance and proportion of the top 10 most abundant phylum, classes, orders, families, and genera in each group. Distinct phylum, classes, orders, families, and genera showed in different colors. The sum of all the other taxon except the top 10 were defined as other.

**Supplementary Figure S5. Co-occurrence profiles and significant correlation among gut bacteria dramatically differ among groups.** Heat map indicating the correlation relationship between differential taxonomic composition. The correlation coefficient is expressed in blue (positive) and red (negative). *, *P*< 0.05; **, *P*< 0.01. Spearman’s correlation.

**Supplementary Figure S6. Gut bacteria prominently discriminative between groups are potentially association with drug treatment or BP modulation. (A)** Venn diagrams show the difference in abundance in different groups. The taxa distinct between the untreated and poor-controlled group are considered as not associated with BP improvement; ARB/ACEI treatment was indicated as not accounting for the discordance between poor-controlled and well-controlled; taxa between untreated vs. treated might be associated with drug consumption; when comparing untreated and well-controlled groups, bacteria linked to BP regulation by ARB/ACEI administration were intended to be identified. Potential taxa crucial for lowering BP by ARB/ACEI were filtrated into 11. Relative abundance of the 11 obtained bacteria in ARB/ACEI treated HTN patients, both well-controlled and poor-controlled, was shown in a heat map.

**Supplementary Figure S7. Metabolic characteristics for the serum metabolome between untreated and ARB/ACEI poor-controlled group. (A)** PCA plots show the metabolic profiles obtained by untargeted GC-TOF/MS data of serum samples from the untreated and poor-controlled group. **(B)** Scatter plots of PLS-DA showing the metabolic discrimination and separation between groups. Significant differences at *P*=6.38e−07 and 0.014 were obtained at Component 1 and Component 2, respectively. **(C)** Scatter plots of OPLS-DA showing the metabolic discrimination and separation between untreated and poor-controlled subjects. **(D)** Serum metabolite detected and the class composition in the untreated and poor-controlled group. **P*<0.05, ***P*<0.01. **(E)** Volcano plots showing the striking alterations of serum metabolites between groups. Metabolites at *P*<0.05 were considered as significantly different. Up, the number of significantly up-regulated serum metabolites in a poor-controlled group compared with the untreated group; Down, the number of dramatically reduced metabolites in the untreated group; None, the number of metabolites between groups that were not significantly different.

**Supplementary Figure S8. Metabolic profiles focusing on the serum metabolome between untreated and ARB/ACEI well-controlled groups. (A)** PCA plots showing the metabolic profiles based on GC-TOF/MS data of serum samples from the untreated and well-controlled group. **(B)** Scatter plots of PLS-DA identifying the metabolic discrimination and separation between groups. Statistic differences of *P*=2.91e−08 and 8.09e−05 between groups were obtained at Component 1 and Component 2, respectively. **(C)** Scatter plots of OPLS-DA displaying the metabolic discrimination and separation between samples in groups. **(D)** Class composition of serum metabolite annotated in the untreated and well-controlled group. **P*<0.05, ***P*<0.01. **(E)** Volcano plots for the prominent shifts of serum metabolites between groups. Metabolites with *P*<0.05 were considered to be significantly different. Up, the number of significantly increased serum metabolites in a well-controlled group compared with the untreated group; Down, the number of dramatically depressed metabolites in the well-controlled group; None, the number of not significantly changed metabolites between groups.

**Supplementary Figure S9. A distinction of metabolic features for the serum metabolome in ARB/ACEI treated patients either poor-controlled or well-controlled. (A)** PCA plots for the metabolic profiles by GC-TOF/MS of serum samples from poor-controlled and well-controlled patients. **(B)** Scatter plots derived from PLS-DA showing the metabolic discrimination and separation between groups. *P*=7.19e−08 and 1.99e−04 were obtained at Component 1 and Component 2, respectively. **(C)** OPLS-DA scatter plots depicting the discrimination and separation of serum metabolic characteristics between groups. **(D)** Relative abundance of serum metabolites and the composition at class level in poor-controlled and well-controlled individuals, respectively. **(E)** Volcano plots showing the discordance of serum metabolites between groups. Metabolites at *P*<0.05 were considered to be significantly disparate. Up, the number of significantly enhanced metabolites in well-controlled group as compared with poor-controlled group; Down, the number of dramatically suppressed metabolites in well-controlled group; None, the number of metabolites not significantly shifted between groups.

**Supplementary Figure S10. Correlation between altered metabolites and fecal microbiota among groups. (A)** Heat map showing the correlation relationship and interactions between the altered taxa and metabolites among groups. *, *P*< 0.05; **, *P*< 0.01, Spearman’s correlation. **(B)** Co-occurrence network of the varied intestinal taxa and metabolites among groups by Spearmen’s correlation analysis. The thresholds derived from the Spearman’s correlation analyses are |r|≥0.3 and *P*≤ 0.05. Circles in red indicate gut bacteria; circles in green indicate the metabolites. The pink lines connecting microbiota and metabolites indicate positive correlation and the blue lines represent negative correlation. The thickness of the line is in proportional to the correlation index of correlation.

**Supplementary Table S1. Baseline characteristics of the participants untreated or treated.**

| **Characteristics** | **Untreated**  **(n=19)** | **Treated**  **(n=36)** | **Poor-controlled**  **(n=12)** | **Well-controlled**  **(n=24)** | ***P*-value**  **( Treated vs. UT)** | ***P*-value**  **( PC vs. UT)** | ***P*-value**  **(WC vs. UT)** | ***P*-value**  **(WC vs. PC)** |
| --- | --- | --- | --- | --- | --- | --- | --- | --- |
| Age, years | 41.00 (35.00-46.00) | 48.50 (43.00-52.00) | 43.50 (40.50-50.00) | 49.00 (44.25-58.75) | 0.002 | 0.173 | 0.053 | 0.001 |
| Male/Female | 19/0 | 35/1 | 12/0 | 23/1 | 1.000 | 1.000 | 1.000 | 1.000 |
| BMI, kg/m^2^ | 29.35 (26.23-31.38) | 28.19 (25.94-31.66) | 30.12 (26.80-32.30) | 27.70 (25.01-31.36) | 0.565 | 0.543 | 0.179 | 0.245 |
| Smoking/no Smoking | 13/6 | 12/24 | 9/3 | 15/9 | 1.000 | 0.511 | 0.755 | 0.709 |
| SBP, mmHg | 153.33 (142.00-161.67) | 133.00 (125.83-142.50) | 147.67 (142.17-159.34) | 128.84 (121.00-134.58) | 0.000 | 0.655 | 0.000 | 0.000 |
| DBP, mmHg | 96.67 (91.33-103.67) | 83.83 (75.33-89.33) | 93.83 (87.75-97.33) | 78.50 (72.67-84.67) | 0.000 | 0.372 | 0.000 | 0.000 |
| FBG, mmol/L | 7.00 (5.87-7.80) | 5.74 (5.05-6.72) | 7.08 (6.73-7.73) | 7.03 (6.41-8.09) | 0.211 | 0.406 | 0.931 | 0.596 |
| Total cholesterol, mmol/L | 5.15 (4.59-6.74) | 4.85 (4.50-5.93) | 5.83 (4.90-6.88) | 5.72 (5.05-6.57) | 0.431 | 0.903 | 0.958 | 0.220 |
| Triglyceride, mmol/L | 5.01 (4.01-5.75) | 2.15 (1.58-3.91) | 4.66 (4.29-5.24) | 5.18 (4.76-6.03) | 0.568 | 0.240 | 0.187 | 0.240 |
| HDLC, mmol/L | 0.95 (0.80-1.07) | 1.05 (0.90-1.14) | 0.90 (0.76-1.10) | 1.08 (0.94-1.18) | 0.670 | 0.256 | 0.047 | 0.047 |
| LDLC, mmol/L | 2.01 (1.52-2.61) | 2.80 (2.18-3.51) | 2.58 (1.56-6.16) | 2.10 (1.58-3.18) | 0.211 | 0.699 | 0.281 | 0.950 |
| Creatinine, umol/L | 70.00 (65.20-74.00) | 70.00 (65.20-74.00) | 72.70 (65.40-75.90) | 64.90 (62.70-70.90) | 0.526 | 0.417 | 0.094 | 0.136 |
| WBC, uL | 7.00 (5.87-7.80) | 7.03 (6.51-8.08) | 7.08 (6.73-7.73) | 7.03 (6.41-8.09) | 0.538 | 0.598 | 0.149 | 0.448 |

UT, untreated; BMI, body mass index; SBP, systolic blood pressure; DBP, diastolic blood pressure; FBG, fasting blood glucose; HDLC, high-density lipoprotein cholesterol; LDLC, low-density lipoprotein cholesterol; WBC, white blood cell. *P* values are obtained from Wilcoxon test.
